# Relationships between circulating metabolites and facial skin aging: a Mendelian randomization study

**DOI:** 10.1186/s40246-023-00470-y

**Published:** 2023-03-17

**Authors:** Zhengye Liu, Jiarui Mi, Huiling Wu

**Affiliations:** 1 Department of Plastic and Aesthetic Center, The First Affiliated Hospital, College of Medicine, Zhejiang University, Hangzhou, Zhejiang China; 2 Department of Cell and Molecular Biology, Karolinska Institutet, Stockholm, Sweden

**Keywords:** Circulating metabolites, Mendelian randomization, Facial skin aging, Unsaturated fatty acids

## Abstract

**Background:**

Blood metabolites are important to various aspects of our health. However, currently, there is little evidence about the role of circulating metabolites in the process of skin aging.

**Objectives:**

To examine the potential effects of circulating metabolites on the process of skin aging.

**Method:**

In the primary analyses, we applied several MR methods to study the associations between 249 metabolites and facial skin aging risk. In the secondary analyses, we replicated the analyses with another array of datasets including 123 metabolites. MR Bayesian model averaging (MR-BMA) method was further used to prioritize the metabolites for the identification of predominant metabolites that are associated with skin aging.

**Results:**

In the primary analyses, only the unsaturation degree of fatty acids was found significantly associated with skin aging with the IVW method after multiple testing (odds ratio = 1.084, 95% confidence interval = 1.049–1.120, *p* = 1.737 × 10^−06^). Additionally, 11 out of 17 unsaturation-related biomarkers showed a significant or suggestively significant causal effect [*p* < 0.05 and > 2 × 10^−4^ (0.05/249 metabolites)]. In the secondary analyses, seven metabolic biomarkers were found significantly associated with skin aging [*p* < 4 × 10^−4^ (0.05/123)], while six of them were related to the unsaturation degree. MR-BMA method validated that the unsaturation degree of fatty acids plays a dominant role in facial skin aging.

**Conclusions:**

Our study used systemic MR analyses and provided a comprehensive atlas for the associations between circulating metabolites and the risk of facial skin aging. Genetically proxied unsaturation degree of fatty acids was highlighted as a dominant factor correlated with the risk of facial skin aging.

**Supplementary Information:**

The online version contains supplementary material available at 10.1186/s40246-023-00470-y.

## Introduction

The skin is the largest organ, covering the entire surface of the human body [1]. Exploring the mechanisms involved in skin aging, especially facial aging, has been an area of interest, not only for esthetic purposes but also because they may provide mechanistic insights into diseases with similar mechanisms [2, 3]. The process of skin aging is affected by circulating metabolites which are involved in a variety of cellular processes including cellular organization, post-translational modification as well as epigenetic changes [4, 5]. Some metabolic biomarkers, such as the levels of unsaturated lipids and polyunsaturated fatty acids (PUFAs), have been reported to play an important role in skin aging [6–10]. However, there are conflicting opinions from different studies [11]. For example, a recent study based on mouse models found that PUFA supplementation can protect mice from photoaging [9]. However, another study reported that PUFA supplementation may induce inflammation and extracellular matrix degradation [10]. There are several reasons that may lead to the contradictory results. Firstly, some studies have been performed on cultured cells or on mouse models, where the conditions may differ from the real-life skin aging process [9, 10]. Furthermore, randomized controlled trials are not an option due to ethical considerations and feasibility. Therefore, most of the studies are based on observational designs, in which the influence of confounding factors or reverse causality cannot be completely ruled out [12]. Finally, circulating metabolites can be divided into multiple subclasses, while different subclasses of metabolites may have distinct effects. Although various types of dietary intake have been studied in different aspects of tissue aging, the overall effect of the complex dietary components is difficult to imply the net effect of individual supplements [13]. Therefore, novel methodologies, which are unbiased by confounders, are needed for further exploration. Due to the increasing popularity of long-term oral supplementation of micronutrients in modern society, it appears as an urgent need for systematic epidemiological evaluation demonstrating the overall impact of metabolites on skin aging.

Mendelian randomization (MR) is an epidemiological method that employs genetic variants as instrumental variables to proxy an exposure variable of interest and study the effect of the exposure on a certain outcome [14]. Since single-nucleotide polymorphisms (SNPs) are assigned randomly at conception, they are unlikely to be affected by confounding factors [15]. The bias to reverse causality is also diminished as genetic variants cannot be affected by the development of the outcome traits [15]. Furthermore, the genetic instrumental variables reflect lifetime exposure, making MR an ideal tool to study aging-related topics. Nowadays, with the advent of high-throughput metabolomics, the levels of hundreds of circulating metabolites can be measured simultaneously. Several genome-wide association studies (GWASs) investigating associations between circulating metabolic biomarkers and SNPs were recently published [16–18]. In this study, by integrating the largest human genomic datasets to date, we employed several MR methods to 1) estimate the causal effects of circulating metabolites on the risk of skin aging and 2) prioritize the metabolites that promote skin aging after adjusting for the effects of similar ones.

## Materials and methods

### Study design

In this study, we have used instrumental variables obtained from two different metabolomics quantitative trait loci studies on circulating metabolites for primary and secondary analyses, respectively, to study the roles of plasma metabolites on skin aging. For the primary analyses, the summary-level GWAS datasets of 249 circulating metabolites that were divided into nine major categories were obtained from UK Biobank (unpublished, accessible via MRC IEU OpenGWAS database). Skin aging-related GWAS datasets were obtained from a recent publication by Roberts V. et al. [3]. For the secondary analyses, summary-level statistics on 123 circulating metabolites were obtained from Kettunen et al. [18] and two-sample MR analyses were performed to further validate our findings. Considering that the metabolites in the same subcategory were highly correlated, we performed an MR Bayesian model averaging (MR-BMA) analysis to prioritize the effect of major metabolites [19]. Only individuals of European ancestry were included in the analyses. Written informed consent and approval from the local ethical committee were obtained by all included studies.

## Data sources

### Metabolic profile for primary analyses

Summary-level datasets on 249 circulating metabolites used in primary analysis were obtained from Nightingale Health Metabolic Biomarkers Phase 1 release study in UK Biobank (June 2019–April 2020) (Table 1). This study included 115,078 randomly selected participants. Metabolic biomarkers were measured with non-fasting baseline EDTA plasma samples by high-throughput nuclear magnetic resonance (NMR) (https://biobank.ndph.ox.ac.uk/ukb/label.cgi?id=220). The biomarkers include 168 absolute metabolites (unit, mmol/L) and 81 metabolite ratios spanning multiple metabolic pathways such as lipoproteins, fatty acids, amino acids, and ketone bodies. The details of sample collection and NMR profiling have been depicted in previous publications [20–22].

**Table 1:** Detailed information of included data sources

Traits	Sample size	Year	Population	PubMed ID	Web source
249 Circulating metabolites (primary analyses)	115,078	2020	European	NA	https://www.ukbiobank.ac.uk/
123 Circulating metabolites (secondary analyses)	24,925	2016	European	27005,778	http://www.computationalmedicine.fi/data/NMR_GWAS/
Facial skin aging (perceived age)	423,992 (8,630 reported looking older than their biological age, 103,300 reported looking about their age, and 312,062 reported looking younger)	2020	European	32339537	https://doi.org/10.5523/bris.21crwsnj4xwjm2g4qi8chathha

BOLT-LMM (linear mixed model) was used to account for population structure, with further adjustment for age, sex, fasting status, and genotyping chips. Over 12.3 million SNPs were included for further analyses after adjusting for covariates and quality control.

### Metabolic profile for secondary analyses

Summary-level datasets on 123 circulating metabolites used in the secondary analysis were obtained from a previous publication by Kuttunen et al. [18] (Table 1). Metabolite concentrations were quantified with high-throughput NMR spectroscopy from 10 studies including 24,925 individuals of European ancestry. Datasets from different cohorts were analyzed separately with an additive model and then pooled together by a fixed-effect meta-analysis, with up to 12,133,295 SNPs included. All metabolite concentrations were adjusted for age, sex, time from the last meal, and ten first principal components.

#### IV selection

SNPs associated with metabolite biomarkers were selected with a conventional genome-wide association significance threshold (*p* < 5 × 10^−8^). Linkage disequilibrium (LD) clumping was used to identify and exclude SNPs that were in LD (*R*^2^ > 0.001 or within ± 10,000 kilobase (kb) distance 1000 Genomes European-ancestry Reference Panel). Mean *F*-statistics were calculated to test for weak instruments as previously described [23].

### Facial skin aging

Summary statistics of skin aging were obtained from a previous GWAS based on UK Biobank [3] (Table 1). Eligible participants identified from health records in National Health Service were invited to participate in baseline assessments including questionnaires, physical measurement, biological samples collection, and follow-ups. The participants were asked the following questions in the questionnaires: “Do people say that you look…?” The possible answers were “Younger than you are,” “Older than you are,” “About your age,” “Do not know,” or “Prefer not to answer.” Participants that did not respond were excluded from subsequent analyses. After imputation and quality control, genome-wide analysis was performed with a linear mixed model using BOLT-LMM. Only individuals of European ancestry were included in the GWAS.

### Mendelian randomization

The inverse-variance weighted (IVW) was used as the main method for causal estimation. Wald ratios of individual SNPs’ effects on the outcome were combined with a fixed-effect IVW when IVs ≤ 3 or a random effect IVW when over 3 IVs were included. Heterogeneities of the IVW analyses were estimated with Cochran’s Q values, I^2^, and the H-statistics [24, 25]. We further performed MR-Egger, weighted median as sensitivity analyses [26–28]. MR-Egger is a method that can give valid causal estimates even with the existence of pleiotropy (*p* for intercept < 0.05), as it detects and corrects for potential horizontal pleiotropy [26]. The weighted median is a method that can be used to strengthen the causal estimates when up to fifty percent of the weight in the MR analyses came from invalid instrument variables [27]. Multivariable Mendelian randomization (MVMR) is a method that estimates the direct effect of different exposures on the outcome after adjusting for the effects of other exposures. In this study, we have also used the MVMR method to estimate the causal associations of candidate metabolites/ratio index on the risk of facial skin aging adjusting for several common risk factors of aging including BMI, smoking behavior (cigarettes per day), and alcohol drinking (alcoholic drinks per week) [29].

### Colocalization analysis

We have also performed a colocalization analysis between the degree of unsaturation and facial skin aging with HyprColoc (R package hyprcoloc: https://rdrr.io/github/jrs95/hyprcoloc/) [30]. The default prior probability that an SNP is causal to one trait was 1 × 10^−4^. If the posterior probability of one SNP being shared between the two traits in one region was greater than 0.8, we regarded it as a signal of colocalization.

### MR Bayesian model averaging (MR-BMA)

As many metabolic traits involved in the study are highly correlated based on sharing a substantial number of SNPs, it appears necessary to correct for the effects of “measured pleiotropy.” Here, we used the MR-BMA to discover the metabolic biomarkers that play predominant roles in the causal associations with skin aging, from a group of related factors [19]. Compared with conventional multivariable MR methods, the MR-BMA method is useful in disentangling the correlated metabolic biomarkers which may act via the same causal pathway [31]. In this study, we followed up the results from primary analyses, by assessing the causal effects of unsaturation-related biomarkers on skin aging with MR-BMA. SNPs associated with all selected biomarkers were pooled and then strictly clumped to exclude SNPs in LD (*R*^2^ < 0.001 in 10,000 kb distance in 1000 Genomes European-ancestry Reference Panel). Posterior probability (PP) was calculated for all specific models (i.e., one biomarker or a combination of multiple biomarkers). The marginal inclusion probability (MIP) for each biomarker, which is the sum of the PP over all models where the biomarker is present, was used to rank the causal associations of the traits with the outcome. We also calculated model-averaged causal effects (MACE), which demonstrates the direct causal effect of a biomarker on skin aging averaged across all related models. Cook’s distance was used to identify outliers in the MR-BMA analyses.

### Statistical analyses

All statistical analyses in this study are two-sided. For primary analyses, a *p*-value < 2 × 10^−4^ (0.05/249, Bonferroni adjusted) was considered statistically significant, and a *p*-value between 0.05 and 2 × 10^−4^ was considered suggestively significant. For secondary analyses, a *p*-value < 4 × 10^−4^ (0.05/123, Bonferroni adjusted) was considered statistically significant, and a p-value between 0.05 and 4 × 10^−4^ was considered suggestively significant. All the analyses were performed on R platform (version 4.1.0), with “TwoSampleMR” (0.5.5), “Mendelian randomization” (0.5.0), “MVMR,” “HyprColoc,” and “ggplot2” packages [28–30, 32, 33].

## Results

### Primary analyses

**Fig. 1:**
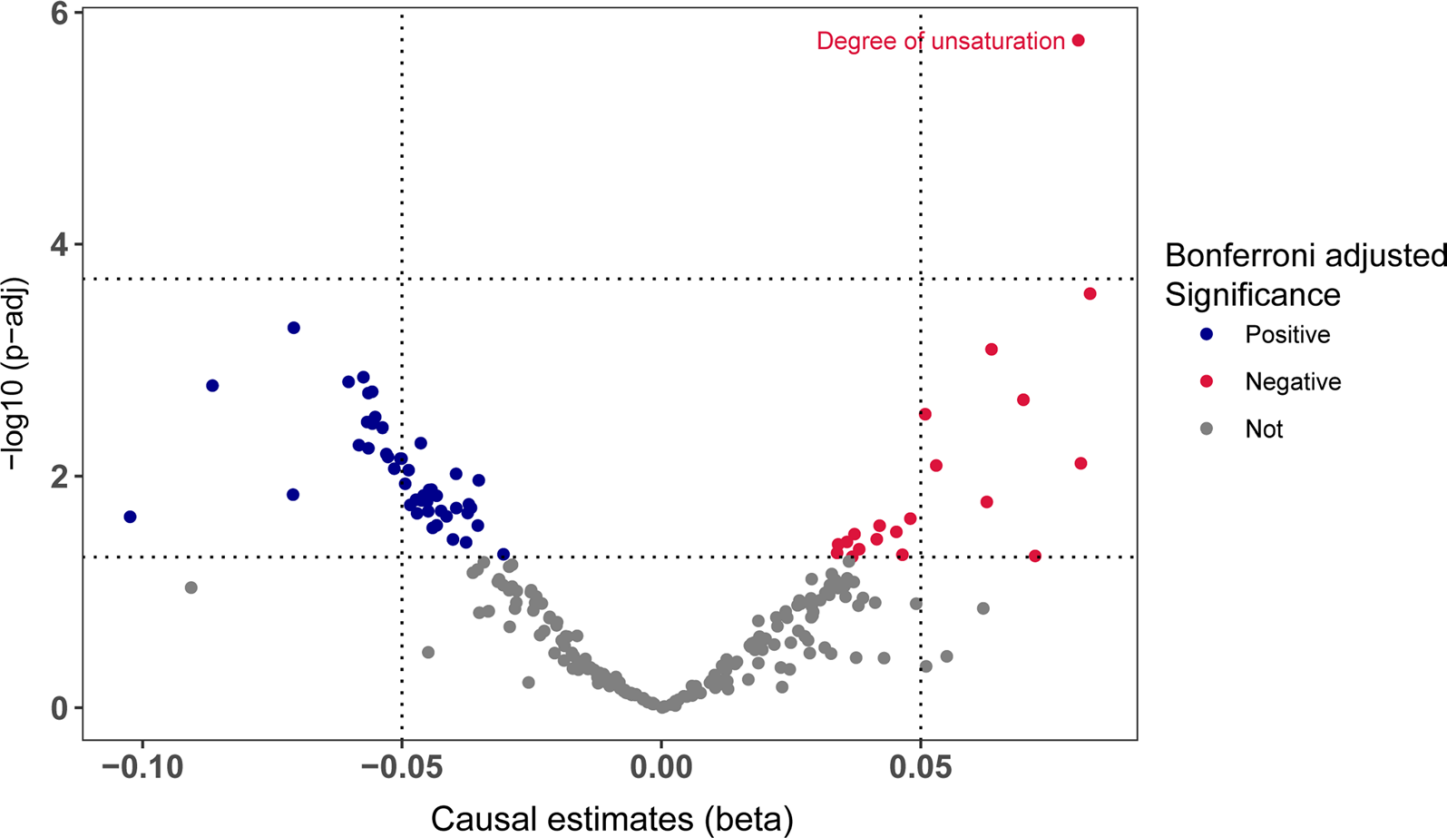
Volcano plot showing the causal estimates of 249 metabolic biomarkers on facial skin aging in the primary analyses with IVW method. IVW, inverse-variance weighted; VLDL, very-low-density lipoprotein; HDL, High-density lipoprotein

By assessing the 249 metabolic biomarkers’ effect on skin aging with univariable MR analyses, only the unsaturation degree of fatty acids was observed to have a significant causal effect on skin aging after Bonferroni adjustment (odds ratio [OR] = 1.084, 95% confidence interval [CI] = 1.049–1.120, *p* = 1.737 × 10^−06^) (Fig. 1, Additional file 2: Table S1). The causal estimate remained consistent with sensitivity analyses (Additional file 2: Tables S2, S3). No horizontal pleiotropy was identified with the MR-Egger method (Additional file 2: Table S4).

**Fig. 2:**
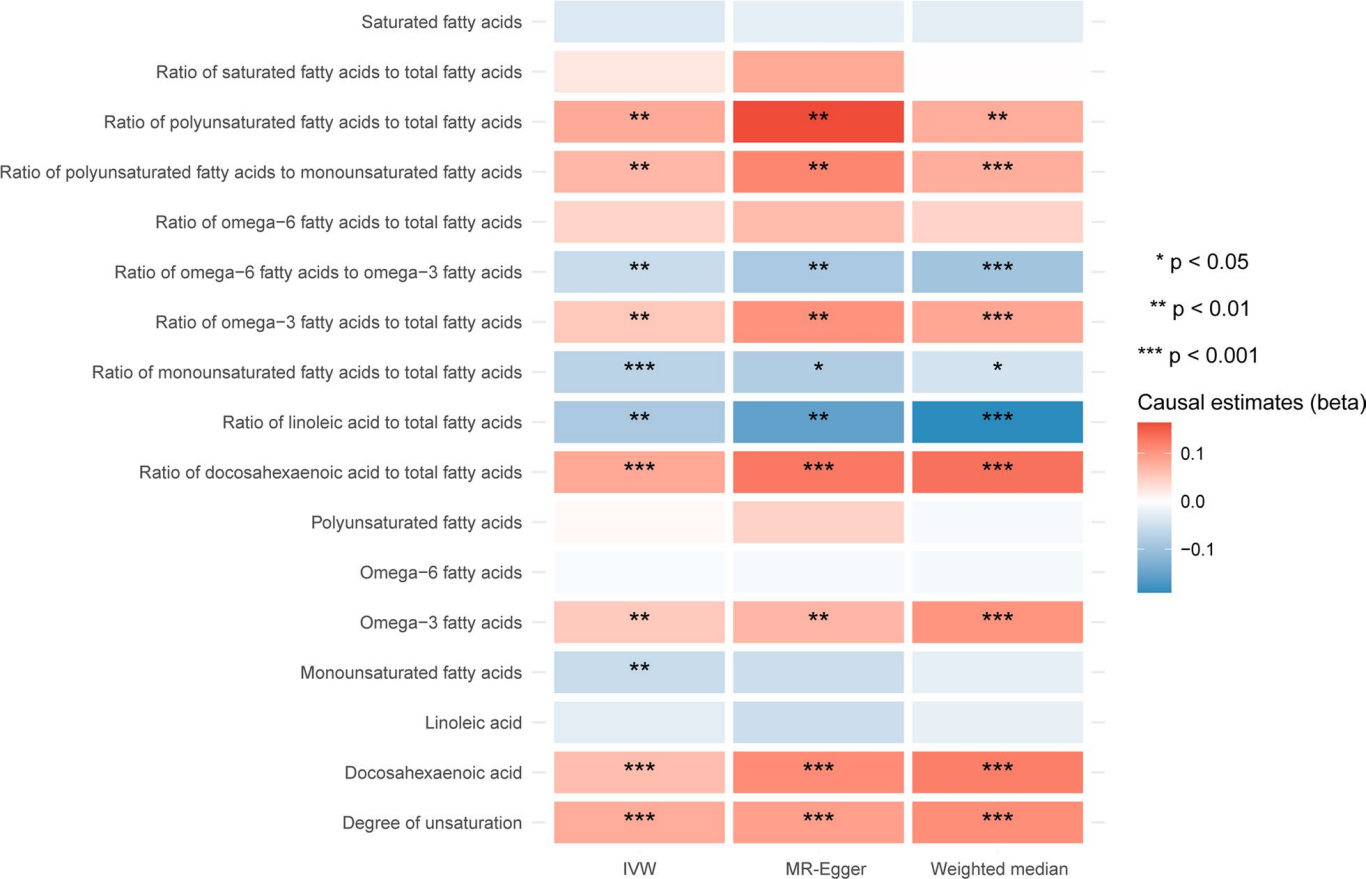
Heatmap showing the causal estimates of unsaturation-related traits on facial skin aging in the primary analyses with IVW, MR-Egger, and weighted median methods. MR, Mendelian randomization; IVW, inverse-variance weighted

Besides, 65 biomarkers were shown to have a suggestively causal effect on skin aging (Additional file 2: Table S1). To understand the relationships between different kinds of metabolic biomarkers and skin aging, we classified the 249 metabolic traits into nine major groups (Fig. 2, Additional file 1: Figs. S1–S8). Among different groups of biomarkers, we surprisingly found that unsaturation-related biomarkers showed a consistent association with skin aging, with 11 out of 17 unsaturation-related biomarkers showing a significant or suggestively significant causal effect (Fig. 2). Among them, the ratio of PUFA to total fatty acids (OR = 1.084, 95% CI 1.022–1.151, *p* = 0.008), PUFA to monounsaturated fatty acids (MUFA) (OR = 1.072, 95% CI 1.025–1.121, *p* = 0.002), n-3 PUFA to fatty acids (OR = 1.054, 95% CI 1.014–1.100, *p* = 0.008), docosahexaenoic acid (DHA) to total fatty acids (OR = 1.086, 95% CI 1.039–1.135, *p* = 2.679 × 10^−04^), n-3 PUFA (OR = 1.052, 95% CI 1.017–1.088, *p* = 0.003), and DHA levels (OR = 1.066, 95% CI 1.027–1.106, *p* = 8.103 × 10^−04^), as biomarkers known to be associated higher unsaturation degree, increased the risk of skin aging (Fig. 2). But the ratio of n-6 to n-3 PUFA (OR = 0.945, 95% CI 0.912–0.979, *p* = 0.002), MUFA to total fatty acids (OR = 0.932, 95% CI 0.895–0.970, *p* = 5.280), linoleic acid to total fatty acids (OR = 0.917, 95% CI 0.869–0.968, *p* = 0.002), and MUFA levels (OR = 0.945, 95% CI 0.908–0.984, *p* = 0.006) were negatively associated with the susceptibility to skin aging (Fig. 2).

Heatmaps of the causal associations are shown in Additional file 1: Figs. S1–S8. Notably, multiple triglyceride-related biomarkers showed an overall tendency to reduce the risk of skin aging; however, none of them remained significant in the sensitivity analyses (Additional file 1: Fig. S1).

Mean F-statistics of all metabolites were higher than 10, indicating a low risk of weak instrument bias. Heterogeneity and horizontal pleiotropy for all the analyses are presented in Additional file 2: Tables S4 and S5. Detailed information on used SNPs is provided in Additional file 2: Table S6.

To rule out the possibility that the skin aging process changes the levels of candidate metabolite or saturation degree, we also performed a reverse MR assessing the causal effects of facial skin aging on the 249 metabolic biomarkers. We observed no significant effects of facial skin aging on any of the included biomarkers with the IVW method (Additional file 2: Table S15).

We further performed a colocalization analysis to test whether the degree of unsaturation colocalizes with facial skin aging, and we identified potential colocalization of the two traits at two regions. One candidate causal SNP rs13107325 is in region Chr4:102688709-103688709, in gene SLC39A8, with a posterior probability of 0.887 and regional probability of 0.897. The other candidate causal SNP rs174564 is in region Chr11:60953822-61953822, with a posterior probability of 0.7938 and regional probability of 1. Interestingly, rs174564 is in a protein-encoding gene FADS2 (fatty acid desaturase 2) which encodes an enzyme that regulates unsaturation of fatty acids by introducing double bonds between defined carbons of the fatty acyl chain.

We further used the MVMR method to estimate the causal associations of the degree of unsaturation on the risk of facial skin aging adjusting for common risk factors of aging including BMI, smoking behavior (cigarettes per day), and alcohol drinking (alcoholic drinks per week). All four exposures remained significantly causally associated with facial skin aging after adjusting for other factors (Additional file 2: Table S16).

### Secondary analyses

**Fig. 3:**
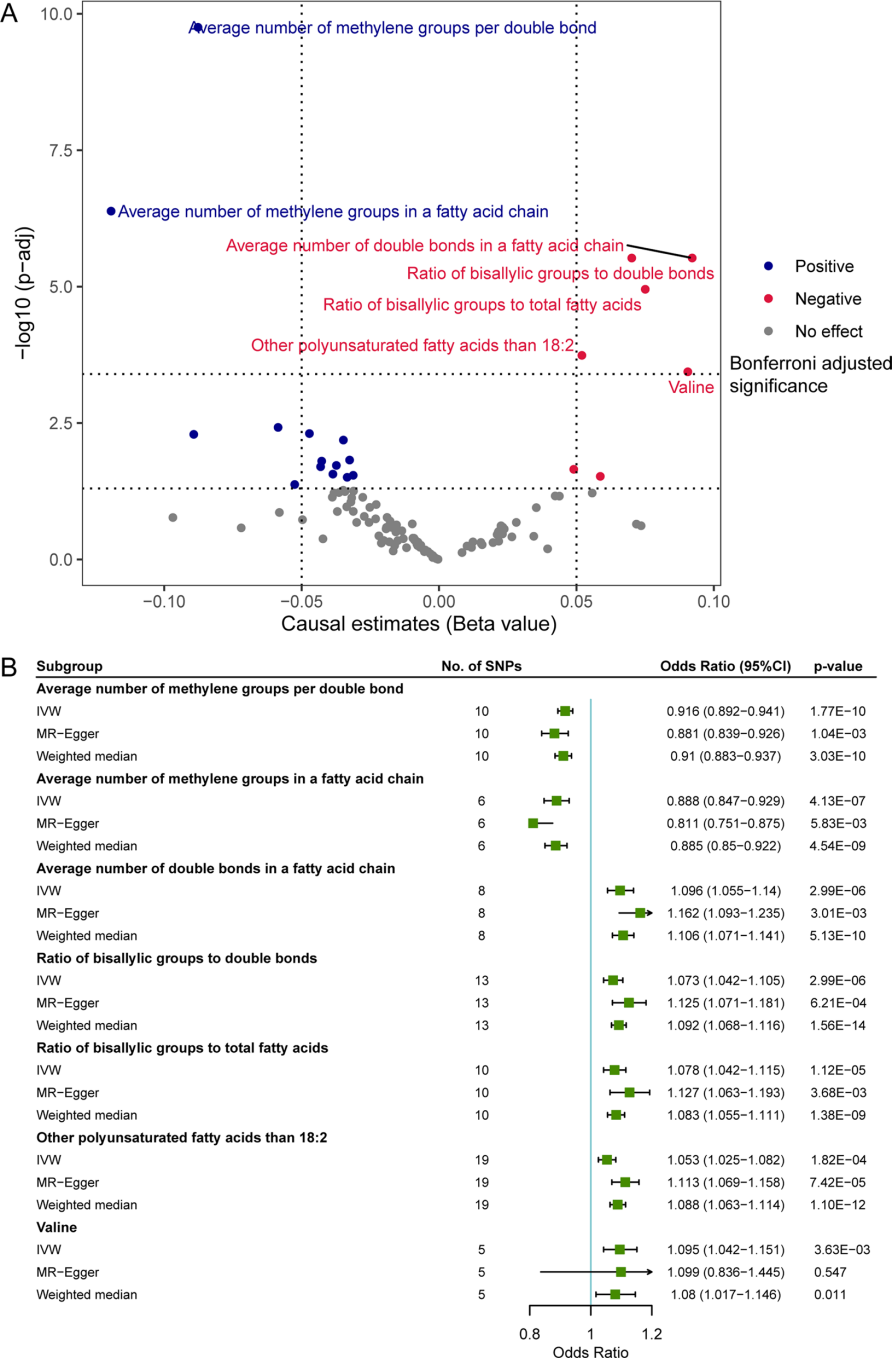
Causal effects of circulating metabolome on facial skin aging in the secondary analyses. **A** Volcano plot showing the causal estimates of 123 metabolic traits on facial skin aging in the secondary analyses with IVW method; **B** forest plots showing the causal estimates of seven metabolic traits that are significantly associated with facial skin aging in the secondary analyses with IVW, MR-Egger, and weighted median methods. MR, Mendelian randomization; IVW, inverse-variance weighted, No., number; SNP, single-nucleotide polymorphism; CI, confidence interval

In the secondary analyses, we estimated the causal effects of 123 circulating metabolic biomarkers on the risk of skin aging with two-sample MR. Seven out of 123 biomarkers demonstrated statistical significance after adjusting for multiple testing (Fig. 3A). Interestingly, six of these seven biomarkers were associated with unsaturation degree of fatty acids (Fig. 3A). Specifically, biomarkers indicative of a higher unsaturation degree, including average number of double bonds in a fatty acid chain (OR = 1.073, 95% CI 1.042–1.105, *p* = 2.99 × 10^−06^), the ratio of bis-allylic groups to double bonds (OR = 1.073, 95% CI 1.042–1.105, *p* = 2.99 × 10^−06^), ratio of bis-allylic groups to total fatty acids (OR = 1.078, 95% CI 1.042–1.115, *p* = 1.12 × 10^−05^), and other polyunsaturated fatty acids than 18:2 (OR = 1.053, 95% CI 1.025–1.082, *p* = 1.82 × 10^−04^), significantly increased the risk of facial skin aging (Fig. 3A, B). On the contrary, biomarkers that lead to a reduced level of unsaturation, including the average number of methylene groups per double bond (OR = 0.916, 95% CI 0.892–0.941, *p* = 1.77 × 10^−10^) and the average number of methylene groups in a fatty acid chain (OR = 0.888, 95% CI 0.847–0.929, *p* = 4.13 × 10^−07^), were inversely correlated with skin aging predisposition (Fig. 3a, b). The significance of the unsaturation-related traits remained consistent in all the sensitivity analyses (Additional file 1: Fig. S9). Causal estimates from sensitivity analyses, horizontal pleiotropy, and heterogeneity are shown in Additional file 2: Tables S7–S11. Detailed information on all included IVs is presented in Additional file 2: Table S12.

### MR Bayesian model averaging

We further performed an MR Bayesian model averaging analysis with 17 unsaturation-related traits from the primary analyses. A total of 463 SNPs were identified as associated with the 17 biomarkers after removing duplicate SNPs. We then removed SNPs in LD and retained 214 SNPs for downstream analyses (Additional file 2: Table S13).

In the MR-BMA analyses, we selected the best models with the highest posterior probability (Additional file 2: Table S14). After that, the MIPs of all included metabolite biomarkers were calculated and used to rank the biomarkers for their causal associations with skin aging risk (Table 2). Degree of unsaturation was identified as the top-ranked biomarker that increases the risk of skin aging (MIP = 0.654, average effect = 0.056, *p* = 0.010). Besides, the ratio of PUFA to MUFA (MIP = 0.106, average effect = 0.008, *p* = 0.040) and MUFA percentage (MIP = 0.093, average effect = -0.006, *p* = 0.040) were also identified to be independently associated with skin aging (Table 2). No outliers were identified in the analyses by using Cook’s distance (Additional file 1: Figures S10–S12). We have used the *Q*-statistics for identifying outliers in MR-BMA; after removing outliers, degree of unsaturation remained the top-ranked biomarker associated with the risk of skin aging (MIP = 0.852, average effect = 0.073, *p* = 0.010) (Additional file 1: Fig. S13).

**Table 2:** Ranking of unsaturation-related metabolic biomarkers for the risk of skin aging using MR-BMA

Metabolite biomarkers	MR-base ID	Ranking by MIP	MIP	Average effect	*p* value
Degree of unsaturation	met-d-Unsaturation	1	0.654	0.056	0.009901
Ratio of polyunsaturated fatty acids to monounsaturated fatty acids	met-d-PUFA_by_MUFA	2	0.106	0.008	0.039604
Ratio of monounsaturated fatty acids to total fatty acids	met-d-MUFA_pct	3	0.093	− 0.006	0.039604
Ratio of polyunsaturated fatty acids to total fatty acids	met-d-PUFA_pct	4	0.088	0.007	0.09901
Ratio of docosahexaenoic acid to total fatty acids	met-d-DHA_pct	5	0.067	0.002	0.366337
Docosahexaenoic acid	met-d-DHA	6	0.025	− 0.001	0.930693
Ratio of omega-3 fatty acids to total fatty acids	met-d-Omega_3_pct	7	0.02	0.002	0.930693
Ratio of linoleic acid to total fatty acids	met-d-LA_pct	8	0.018	− 0.001	0.960396
Ratio of omega-6 fatty acids to total fatty acids	met-d-Omega_6_pct	9	0.017	0.001	0.970297
Ratio of omega-6 fatty acids to omega-3 fatty acids	met-d-Omega_6_by_Omega_3	10	0.016	0.001	0.950495
Linoleic acid	met-d-LA	11	0.013	− 0.002	0.980198
Monounsaturated fatty acids	met-d-MUFA	12	0.012	− 0.001	0.039604
Omega-6 fatty acids	met-d-Omega_6	13	0.011	0.001	0.980198
Omega-3 fatty acids	met-d-Omega_3	14	0.011	0	0.970297
Saturated fatty acids	met-d-SFA	15	0.008	0	0.990099
Polyunsaturated fatty acids	met-d-PUFA	16	0.007	0	0.990099
Ratio of saturated fatty acids to total fatty acids	met-d-SFA_pct	17	0.005	0	1

*MIP* marginal inclusion probability, *MR* Mendelian randomization, *MR-BMA* MR based on Bayesian model averaging, *PUFA* polyunsaturated fatty acids, *MUFA* monounsaturated fatty acids, *DHA* docosahexaenoic acid, *LA* linoleic acid, SFA saturated fatty acids

## Discussion

The aging process may be influenced by various factors including intrinsic aging, environment, and lifestyle habits [34–37]. Nutritional factors have been shown to play an important role in maintaining the normal function of the skin [38]. However, the association between nutritional status and changes in skin appearance remains unclear. Our study, for the first time, comprehensively studied the individual causal effects of a broad range of circulating metabolic traits on the predisposition of skin aging. Our results highlighted the effect of the degree of unsaturation and several unsaturation-related metabolites on the risk of skin aging. We also observed that multiple triglyceride-related biomarkers showed a trend toward reduced skin aging risk. This further confirmed the robustness of our analyses as triglycerides are a major component of sebum, which is known to be important for moisturizing and protecting human skin [39].

There are limited publications on unsaturated fatty acids in skin aging. Some studies found an improved photoprotection with dietary supplementation of PUFAs [12, 40–42]. However, most of these studies were based on observational designs or short-term oral supplementation and only assessed the interaction of unsaturation with environmental risk factors for skin aging such as sunlight exposure. The long-term effects of lipid unsaturation degree on skin aging process are still not fully established. Besides, oral supplementation commonly contains several categories of unsaturated fatty acids, while fatty acids with different degrees of unsaturation may generate diverse effects on skin aging [9, 42]. Even though shown to be photoprotective, fat unsaturation has also been reported to be associated with aging by several studies [9, 43, 44]. A higher degree of fat unsaturation in tissue membrane promotes the aging process through free radical production and oxidative stress [45, 46]. This is consistent with our observations in this study that genetically proxied degree of unsaturation was positively correlated with the risk of facial skin aging as an independent risk factor (Fig. 1). We also observed that increased ratios of total PUFA, n-3 PUFA, and DHA tend to contribute to skin aging, while a higher ratio of n-6 to n-3 PUFA, and the ratio of linoleic acid, reduced the risk of skin aging (Fig. 1). Intriguingly, neither the absolute level nor the percentage of saturated fatty acids were associated with facial skin aging in any of the methods of analysis, suggesting that changes in the proportions of different kinds of unsaturated fatty acids with different degrees of unsaturation were more important factors for skin aging (Figs. 1, 2).

These results were validated in our secondary analyses with an independent dataset for circulating metabolites. As n-6 PUFAs have fewer double bonds than n-3 PUFAs (2–4 compared to 3–6 double bonds), we observed that a higher number of double bonds in fatty acids increased facial skin aging risk (Fig. 3). Furthermore, we also found that higher numbers and ratios of bis-allylic groups, which have been reported to determine cells’ susceptibility to free radical-mediated peroxidative events, added to the risk of skin aging (Fig. 3) [47]. DHA is a kind of n-3 PUFA that contains five bis-allylic positions. It is highly sensitive to radical oxidation and may lead to deleterious advanced lipid peroxidation end products (ALEs) [48, 49]. When ALEs are formed at toxic levels, they may disrupt the cellular membrane and cause DNA damage [50, 51]. In this study, we also observed that genetically proxied higher DHA percentage and absolute level had a causal effect on skin aging (Fig. 2). Metabolite biomarkers analyzed in this study include both absolute levels and their percentage in total fatty acids or ratios to other metabolites. Intriguingly, it appears that the percentages of these unsaturation-related metabolites tend to generate a more significant effect on skin aging than their absolute concentrations (Fig. 1, Table 2). Previous publications also suggested that dietary intake of PUFA should be below a ceiling percentage of total energy [52]. The evidence suggests that maintaining a rational proportion of dietary unsaturated fatty acids might be of great importance to prevent their adverse effects on human skin.

The effects of PUFA on aging and obesity have also been intensively studied in mouse models. It has been shown that linoleic acid, compared with saturated fat, is more prone to induce obesity and insulin resistance and reduce motility (14962692, 22334255, 27886622). To rule out the potential confounding factors and mediation effects, we also performed the MVMR to adjust for BMI and common lifestyle habits that could lead to metabolic dysregulation. Notably, our MVMR results further confirmed our MR findings, indicating that the unsaturation degree of fatty acid may be an independent risk factor in inducing facial aging.

There are several strengths and limitations of our study. To our knowledge, this is the first study employing an MR approach to assess the effects of individual circulating metabolites on skin aging. This is particularly important as results from observational studies or experimental mouse models are based on the aggregate effect of different dietary intake components [53]. Our results highlighted the importance of unsaturation degree in facial skin aging and provided a good reference for future studies. Besides, by using genetic variants as instrumental variables for the metabolic biomarkers, we minimized the bias from confounding factors and reverse causality. Also, the results remained consistent across the primary and replication analyses, which guaranteed the robustness of the findings. Lastly, the study population were refined to individuals of European ancestry to minimize bias from population stratification. However, this also restricted the generalizability of the conclusions, and it is necessary to validate the findings in other populations. Another restriction is that using genetic variants as proxies mimics a lifetime exposure, while oral supplementation for short period may generate a different effect. Finally, the metabolic biomarkers were measured in a non-fasting population, which can lead to inaccurate measurements. Nevertheless, the GWASs of the metabolites were adjusted for fasting time and found the alterations in the estimates were neglectable. Lastly, our MR study is unable to explore the cellular and molecular mechanisms underlying the effects of metabolites on facial aging. The gene GPR120 has been identified as the natural receptor of PUFA; we believed that further investigations using mutant mouse models (such as *Gpr120* mutant mice), skin cell in vitro culture, single-cell RNA-seq, and proteomics experiments are warranted to reveal the detailed molecular events in skin tissue after the supplementation of PUFA.

In conclusion, our study provided evidence suggesting the unsaturation degree of circulating fatty acids as the predominant trait that is involved in the development of facial skin aging. Further studies are needed to investigate the role of long-term supplementation of unsaturated fatty acids in facial skin aging.

## Supplementary Information

**Additional file1**: **Figure S1**. Heatmap showing the causal estimates of triglycerides related traits on facial skin aging in the primary analyses with IVW, MR-Egger, and weighted median methods. **Figure S2**. Heatmap showing the causal estimates of amino acids on facial skin aging in the primary analyses with IVW, MR-Egger, and weighted median methods. **Figure S3**. Heatmap showing the causal estimates of cholesterol ester on facial skin aging in the primary analyses with IVW, MR-Egger, and weighted median methods. **Figure S4**. Heatmap showing the causal estimates of free cholesterol on facial skin aging in the primary analyses with IVW, MR-Egger, and weighted median methods. **Figure S5**. Heatmap showing the causal estimates of lipoprotein cholesterol on facial skin aging in the primary analyses with IVW, MR-Egger, and weighted median methods. **Figure S6**: Heatmap showing the causal estimates of small metabolites on facial skin aging in the primary analyses with IVW, MR-Egger, and weighted median methods. **Figure S7**. Heatmap showing the causal estimates of phospholipids on facial skin aging in the primary analyses with IVW, MR-Egger, and weighted median methods. **Figure S8**. Heatmap showing the causal estimates of total lipids on facial skin aging in the primary analyses with IVW, MR-Egger, and weighted median methods. **Figure S9**. Heatmap showing the causal estimates of 123 metabolic traits on facial skin aging in the secondary analyses with IVW, MR-Egger, and weighted median methods. **Figure S10**. Dot plot of Cook’s distance for the causal effects of degree of unsaturation on facial skin aging with MR-BMA method. **Figure S11**. Dot plot of Cook’s distance for the causal effects of MUFA on facial skin aging with MR-BMA method. **Figure S12**. Dot plot of Cook’s distance for the causal effects of PUFA to MUFA ratio on facial skin aging with MR-BMA method. **Figure S13**. Dot plot of Q-statistics for the causal effects of degree of unsaturation on facial skin aging with MR-BMA method.

**Additional file 2**: **Table S1**. Results with IVW method from primary analyses. **Table S2**. Results with MR-Egger method from primary analyses. **Table S3**. Results with weighted median method from primary analyses. **Table S4**. Measurements of horizontal pleiotropy in primary analyses. **Table S5**. Measurements of heterogeneity in primary analyses. **Table S6**. Detailed information of all SNPs in primary analyses. **Table S7**. Results with Wald ratios and IVW method from secondary analyses. **Table S8**. Results with MR-Egger method from secondary analyses. **Table S9**. Results with weighted median method from secondary analyses. **Table S10**. Measurements of horizontal pleiotropy in secondary analyses. **Table S11**. Measurements of heterogeneity in secondary analyses. **Table S12**: Detailed information of all SNPs in secondary analyses. **Table S13**. Detailed information of all SNPs in MR-BMA analyses. **Table S14**. Best models and corresponding posterior probabilities in MR-BMA analyses. **Table S15**. Causal effects of facial skin aging on metabolic traits from primary analyses with IVW method. **Table S16**. Direct effects of common risk factors on facial skin aging with Multivariable MR analyses.

## Data Availability

All data generated or analyzed during this study are included in this published article and its supplementary information files. The summary-level statistics of GWAS dataset for metabolic traits can be accessed through IEU open GWAS project (https://gwas.mrcieu.ac.uk/datasets/) or MR-Base (https://www.mrbase.org/) website under the accession ID met-d and met-c. The summary-level statistics of facial skin aging can be accessed from a previous publication (https://doi.org/10.5523/bris.21crwsnj4xwjm2g4qi8chathha).

## Abbreviations

ALEs: Advanced lipid peroxidation end products
CI: Confidence interval
DHA: Docosahexaenoic acid
DNA: Deoxyribonucleic acid
GWASs: Genome-wide association studies
IVW: Inverse-variance weighted
kb: Kilobase
LD: Linkage disequilibrium
LMM: Linear mixed model
MACE: Model-averaged causal effects
MIP: Marginal inclusion probability
MR: Mendelian randomization
MR-BMA: MR Bayesian model averaging
MUFA: Monounsaturated fatty acids
NMR: Nuclear magnetic resonance
OR: Odds ratio
PP: Posterior probability
PUFA: Polyunsaturated fatty acids
SNP: Single-nucleotide polymorphisms
