# Supplementary figures and images for: Relationships between circulating metabolites and facial skin aging: a Mendelian randomization study
Source: Hum Genomics. 2023 Mar 17;17:23. doi: 10.1186/s40246-023-00470-y (PMC10022075; doi:10.1186/s40246-023-00470-y)

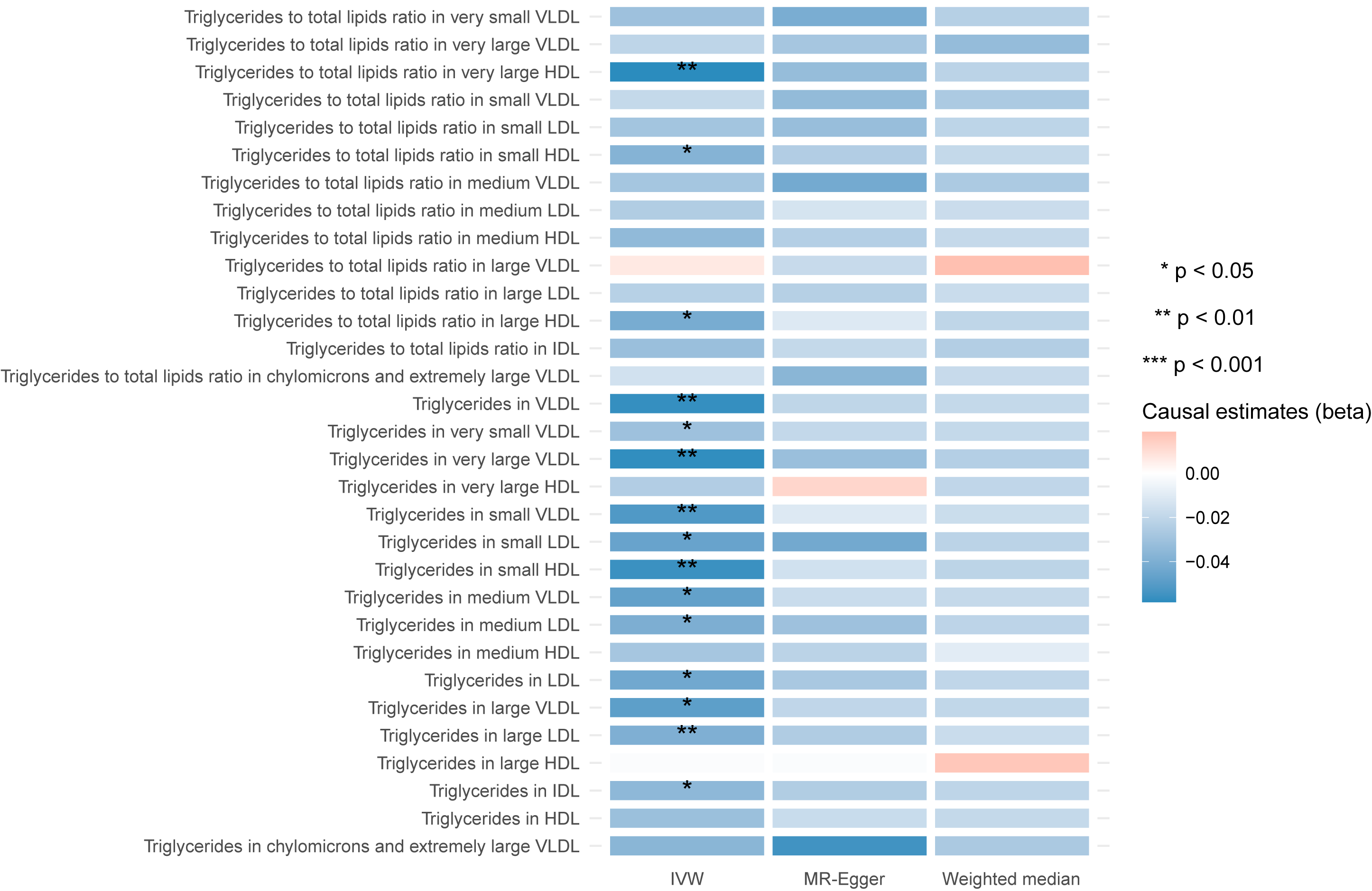

Supplement: Supplementary file 1 — Additional file1: Figure S1. Heatmap showing the causal estimates of triglycerides related traits on facial skin aging in the primary analyses with IVW, MR-Egger, and weighted median methods. Figure S2. Heatmap showing the causal estimates of amino acids on facial skin aging in the primary analyses with IVW, MR-Egger, and weighted median methods. Figure S3. Heatmap showing the causal estimates of cholesterol ester on facial skin aging in the primary analyses with IVW, MR-Egger, and weighted median methods. Figure S4. Heatmap showing the causal estimates of free cholesterol on facial skin aging in the primary analyses with IVW, MR-Egger, and weighted median methods. Figure S5. Heatmap showing the causal estimates of lipoprotein cholesterol on facial skin aging in the primary analyses with IVW, MR-Egger, and weighted median methods. Figure S6: Heatmap showing the causal estimates of small metabolites on facial skin aging in the primary analyses with IVW, MR-Egger, and weighted median methods. Figure S7. Heatmap showing the causal estimates of phospholipids on facial skin aging in the primary analyses with IVW, MR-Egger, and weighted median methods. Figure S8. Heatmap showing the causal estimates of total lipids on facial skin aging in the primary analyses with IVW, MR-Egger, and weighted median methods. Figure S9. Heatmap showing the causal estimates of 123 metabolic traits on facial skin aging in the secondary analyses with IVW, MR-Egger, and weighted median methods. Figure S10. Dot plot of Cook’s distance for the causal effects of degree of unsaturation on facial skin aging with MR-BMA method. Figure S11. Dot plot of Cook’s distance for the causal effects of MUFA on facial skin aging with MR-BMA method. Figure S12. Dot plot of Cook’s distance for the causal effects of PUFA to MUFA ratio on facial skin aging with MR-BMA method. Figure S13. Dot plot of Q-statistics for the causal effects of degree of unsaturation on facial skin aging with MR-BMA method. [file 40246_2023_470_MOESM1_ESM.zip › Supplementary/Supplementary Figure 1. Triglycerides.tif]

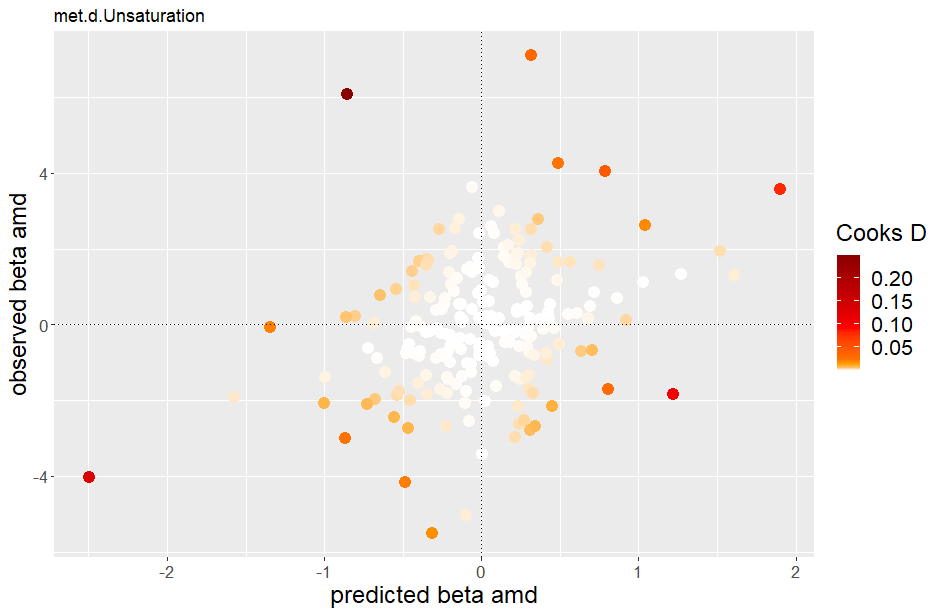

Supplement: Supplementary file 1 — Additional file1: Figure S1. Heatmap showing the causal estimates of triglycerides related traits on facial skin aging in the primary analyses with IVW, MR-Egger, and weighted median methods. Figure S2. Heatmap showing the causal estimates of amino acids on facial skin aging in the primary analyses with IVW, MR-Egger, and weighted median methods. Figure S3. Heatmap showing the causal estimates of cholesterol ester on facial skin aging in the primary analyses with IVW, MR-Egger, and weighted median methods. Figure S4. Heatmap showing the causal estimates of free cholesterol on facial skin aging in the primary analyses with IVW, MR-Egger, and weighted median methods. Figure S5. Heatmap showing the causal estimates of lipoprotein cholesterol on facial skin aging in the primary analyses with IVW, MR-Egger, and weighted median methods. Figure S6: Heatmap showing the causal estimates of small metabolites on facial skin aging in the primary analyses with IVW, MR-Egger, and weighted median methods. Figure S7. Heatmap showing the causal estimates of phospholipids on facial skin aging in the primary analyses with IVW, MR-Egger, and weighted median methods. Figure S8. Heatmap showing the causal estimates of total lipids on facial skin aging in the primary analyses with IVW, MR-Egger, and weighted median methods. Figure S9. Heatmap showing the causal estimates of 123 metabolic traits on facial skin aging in the secondary analyses with IVW, MR-Egger, and weighted median methods. Figure S10. Dot plot of Cook’s distance for the causal effects of degree of unsaturation on facial skin aging with MR-BMA method. Figure S11. Dot plot of Cook’s distance for the causal effects of MUFA on facial skin aging with MR-BMA method. Figure S12. Dot plot of Cook’s distance for the causal effects of PUFA to MUFA ratio on facial skin aging with MR-BMA method. Figure S13. Dot plot of Q-statistics for the causal effects of degree of unsaturation on facial skin aging with MR-BMA method. [file 40246_2023_470_MOESM1_ESM.zip › Supplementary/Supplementary Figure 10 Cooks's distance for unsaturation.tiff]

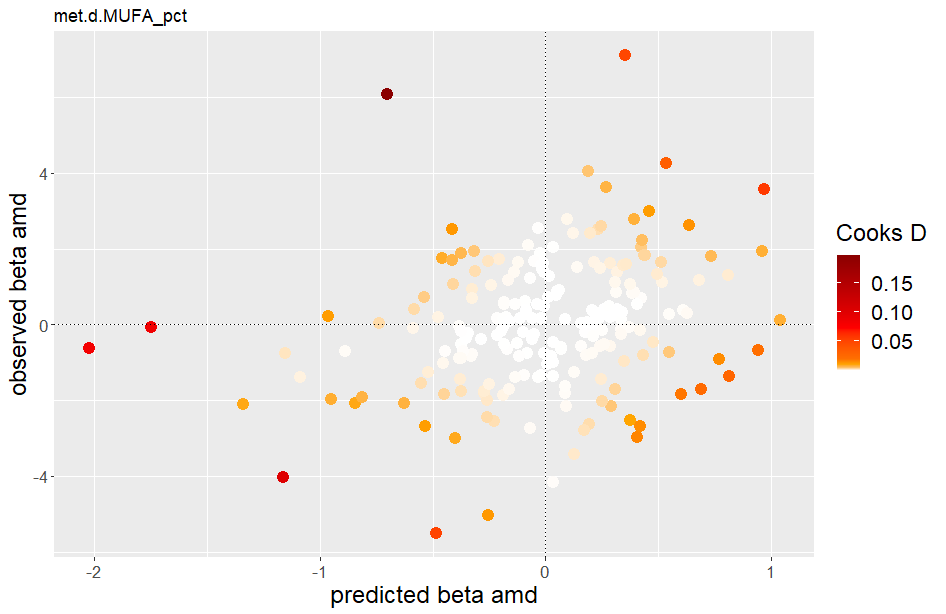

Supplement: Supplementary file 1 — Additional file1: Figure S1. Heatmap showing the causal estimates of triglycerides related traits on facial skin aging in the primary analyses with IVW, MR-Egger, and weighted median methods. Figure S2. Heatmap showing the causal estimates of amino acids on facial skin aging in the primary analyses with IVW, MR-Egger, and weighted median methods. Figure S3. Heatmap showing the causal estimates of cholesterol ester on facial skin aging in the primary analyses with IVW, MR-Egger, and weighted median methods. Figure S4. Heatmap showing the causal estimates of free cholesterol on facial skin aging in the primary analyses with IVW, MR-Egger, and weighted median methods. Figure S5. Heatmap showing the causal estimates of lipoprotein cholesterol on facial skin aging in the primary analyses with IVW, MR-Egger, and weighted median methods. Figure S6: Heatmap showing the causal estimates of small metabolites on facial skin aging in the primary analyses with IVW, MR-Egger, and weighted median methods. Figure S7. Heatmap showing the causal estimates of phospholipids on facial skin aging in the primary analyses with IVW, MR-Egger, and weighted median methods. Figure S8. Heatmap showing the causal estimates of total lipids on facial skin aging in the primary analyses with IVW, MR-Egger, and weighted median methods. Figure S9. Heatmap showing the causal estimates of 123 metabolic traits on facial skin aging in the secondary analyses with IVW, MR-Egger, and weighted median methods. Figure S10. Dot plot of Cook’s distance for the causal effects of degree of unsaturation on facial skin aging with MR-BMA method. Figure S11. Dot plot of Cook’s distance for the causal effects of MUFA on facial skin aging with MR-BMA method. Figure S12. Dot plot of Cook’s distance for the causal effects of PUFA to MUFA ratio on facial skin aging with MR-BMA method. Figure S13. Dot plot of Q-statistics for the causal effects of degree of unsaturation on facial skin aging with MR-BMA method. [file 40246_2023_470_MOESM1_ESM.zip › Supplementary/Supplementary Figure 11 Cooks's distance for MUFA.tiff]

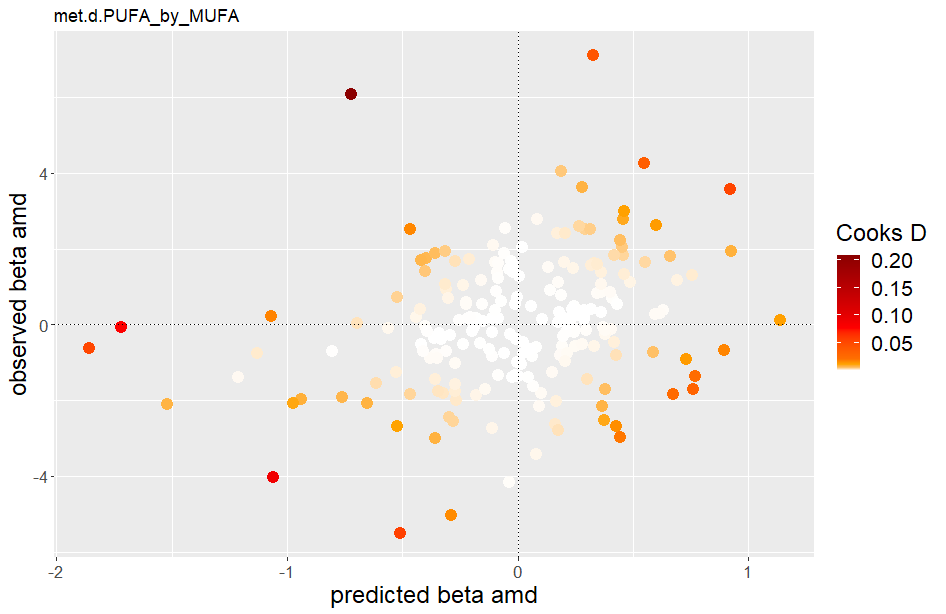

Supplement: Supplementary file 1 — Additional file1: Figure S1. Heatmap showing the causal estimates of triglycerides related traits on facial skin aging in the primary analyses with IVW, MR-Egger, and weighted median methods. Figure S2. Heatmap showing the causal estimates of amino acids on facial skin aging in the primary analyses with IVW, MR-Egger, and weighted median methods. Figure S3. Heatmap showing the causal estimates of cholesterol ester on facial skin aging in the primary analyses with IVW, MR-Egger, and weighted median methods. Figure S4. Heatmap showing the causal estimates of free cholesterol on facial skin aging in the primary analyses with IVW, MR-Egger, and weighted median methods. Figure S5. Heatmap showing the causal estimates of lipoprotein cholesterol on facial skin aging in the primary analyses with IVW, MR-Egger, and weighted median methods. Figure S6: Heatmap showing the causal estimates of small metabolites on facial skin aging in the primary analyses with IVW, MR-Egger, and weighted median methods. Figure S7. Heatmap showing the causal estimates of phospholipids on facial skin aging in the primary analyses with IVW, MR-Egger, and weighted median methods. Figure S8. Heatmap showing the causal estimates of total lipids on facial skin aging in the primary analyses with IVW, MR-Egger, and weighted median methods. Figure S9. Heatmap showing the causal estimates of 123 metabolic traits on facial skin aging in the secondary analyses with IVW, MR-Egger, and weighted median methods. Figure S10. Dot plot of Cook’s distance for the causal effects of degree of unsaturation on facial skin aging with MR-BMA method. Figure S11. Dot plot of Cook’s distance for the causal effects of MUFA on facial skin aging with MR-BMA method. Figure S12. Dot plot of Cook’s distance for the causal effects of PUFA to MUFA ratio on facial skin aging with MR-BMA method. Figure S13. Dot plot of Q-statistics for the causal effects of degree of unsaturation on facial skin aging with MR-BMA method. [file 40246_2023_470_MOESM1_ESM.zip › Supplementary/Supplementary Figure 12 Cooks's distance for PUFA by MUFA.tiff]

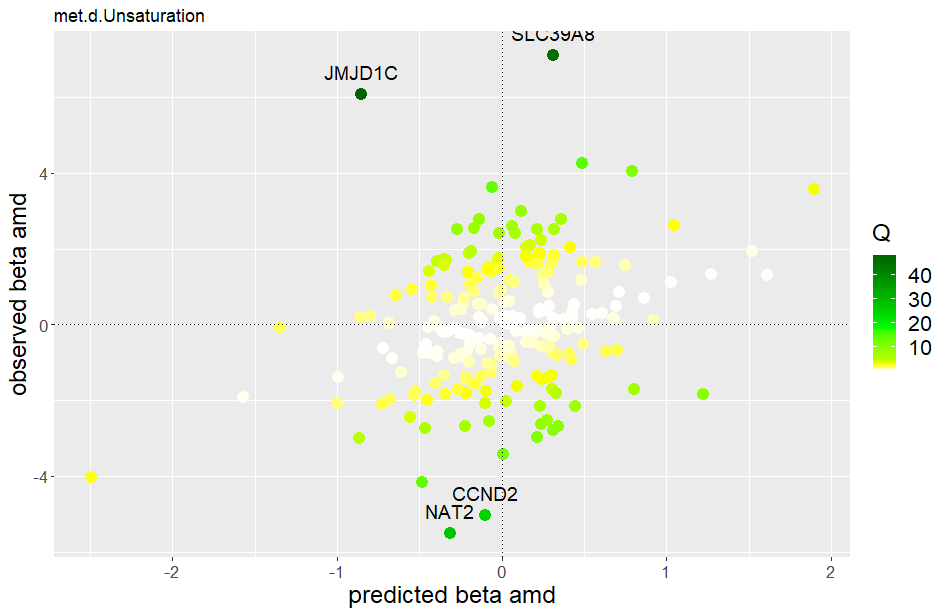

Supplement: Supplementary file 1 — Additional file1: Figure S1. Heatmap showing the causal estimates of triglycerides related traits on facial skin aging in the primary analyses with IVW, MR-Egger, and weighted median methods. Figure S2. Heatmap showing the causal estimates of amino acids on facial skin aging in the primary analyses with IVW, MR-Egger, and weighted median methods. Figure S3. Heatmap showing the causal estimates of cholesterol ester on facial skin aging in the primary analyses with IVW, MR-Egger, and weighted median methods. Figure S4. Heatmap showing the causal estimates of free cholesterol on facial skin aging in the primary analyses with IVW, MR-Egger, and weighted median methods. Figure S5. Heatmap showing the causal estimates of lipoprotein cholesterol on facial skin aging in the primary analyses with IVW, MR-Egger, and weighted median methods. Figure S6: Heatmap showing the causal estimates of small metabolites on facial skin aging in the primary analyses with IVW, MR-Egger, and weighted median methods. Figure S7. Heatmap showing the causal estimates of phospholipids on facial skin aging in the primary analyses with IVW, MR-Egger, and weighted median methods. Figure S8. Heatmap showing the causal estimates of total lipids on facial skin aging in the primary analyses with IVW, MR-Egger, and weighted median methods. Figure S9. Heatmap showing the causal estimates of 123 metabolic traits on facial skin aging in the secondary analyses with IVW, MR-Egger, and weighted median methods. Figure S10. Dot plot of Cook’s distance for the causal effects of degree of unsaturation on facial skin aging with MR-BMA method. Figure S11. Dot plot of Cook’s distance for the causal effects of MUFA on facial skin aging with MR-BMA method. Figure S12. Dot plot of Cook’s distance for the causal effects of PUFA to MUFA ratio on facial skin aging with MR-BMA method. Figure S13. Dot plot of Q-statistics for the causal effects of degree of unsaturation on facial skin aging with MR-BMA method. [file 40246_2023_470_MOESM1_ESM.zip › Supplementary/Supplementary Figure 13 Q statistics.tiff]

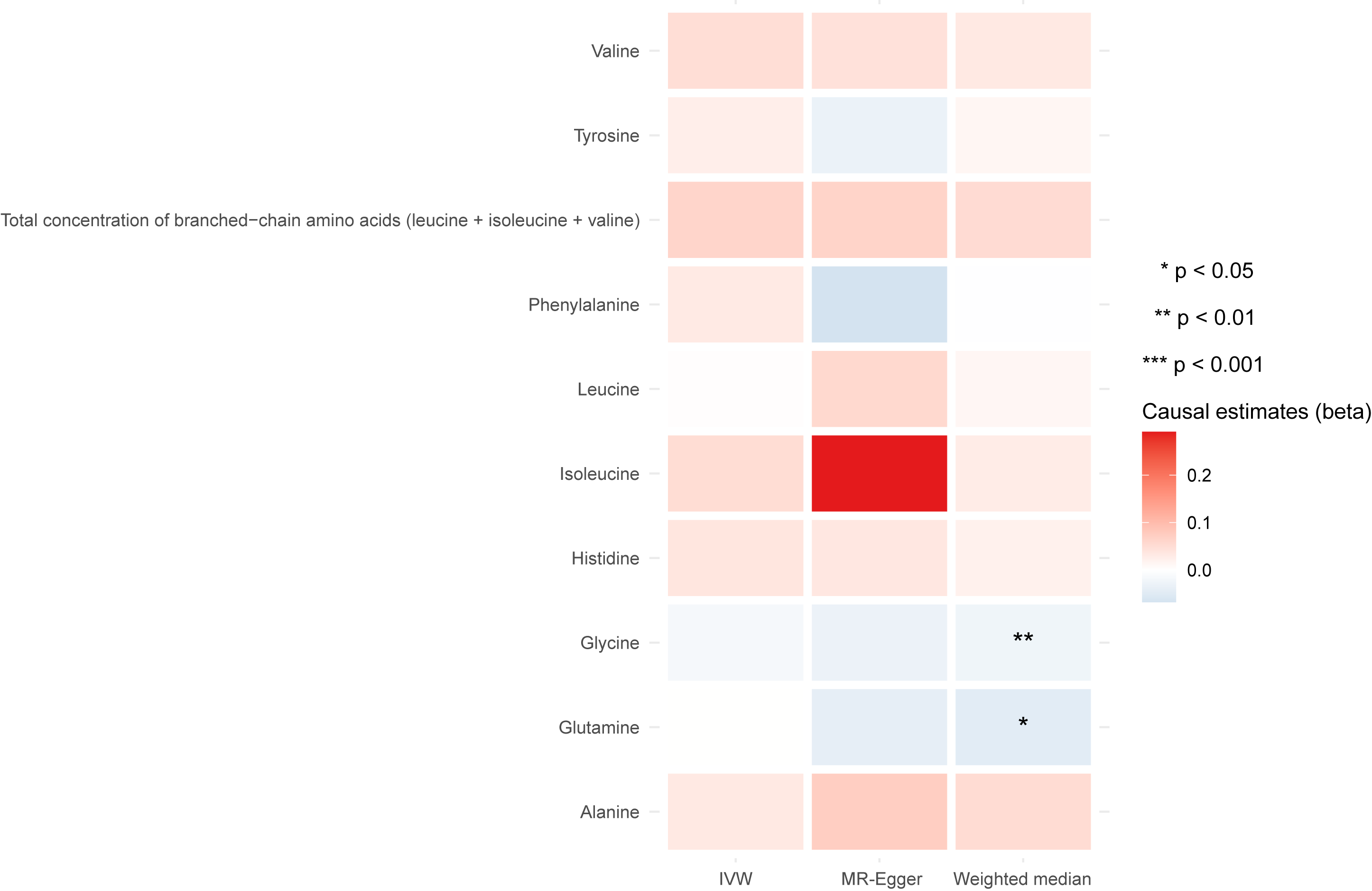

Supplement: Supplementary file 1 — Additional file1: Figure S1. Heatmap showing the causal estimates of triglycerides related traits on facial skin aging in the primary analyses with IVW, MR-Egger, and weighted median methods. Figure S2. Heatmap showing the causal estimates of amino acids on facial skin aging in the primary analyses with IVW, MR-Egger, and weighted median methods. Figure S3. Heatmap showing the causal estimates of cholesterol ester on facial skin aging in the primary analyses with IVW, MR-Egger, and weighted median methods. Figure S4. Heatmap showing the causal estimates of free cholesterol on facial skin aging in the primary analyses with IVW, MR-Egger, and weighted median methods. Figure S5. Heatmap showing the causal estimates of lipoprotein cholesterol on facial skin aging in the primary analyses with IVW, MR-Egger, and weighted median methods. Figure S6: Heatmap showing the causal estimates of small metabolites on facial skin aging in the primary analyses with IVW, MR-Egger, and weighted median methods. Figure S7. Heatmap showing the causal estimates of phospholipids on facial skin aging in the primary analyses with IVW, MR-Egger, and weighted median methods. Figure S8. Heatmap showing the causal estimates of total lipids on facial skin aging in the primary analyses with IVW, MR-Egger, and weighted median methods. Figure S9. Heatmap showing the causal estimates of 123 metabolic traits on facial skin aging in the secondary analyses with IVW, MR-Egger, and weighted median methods. Figure S10. Dot plot of Cook’s distance for the causal effects of degree of unsaturation on facial skin aging with MR-BMA method. Figure S11. Dot plot of Cook’s distance for the causal effects of MUFA on facial skin aging with MR-BMA method. Figure S12. Dot plot of Cook’s distance for the causal effects of PUFA to MUFA ratio on facial skin aging with MR-BMA method. Figure S13. Dot plot of Q-statistics for the causal effects of degree of unsaturation on facial skin aging with MR-BMA method. [file 40246_2023_470_MOESM1_ESM.zip › Supplementary/Supplementary Figure 2. Amino acid.tif]

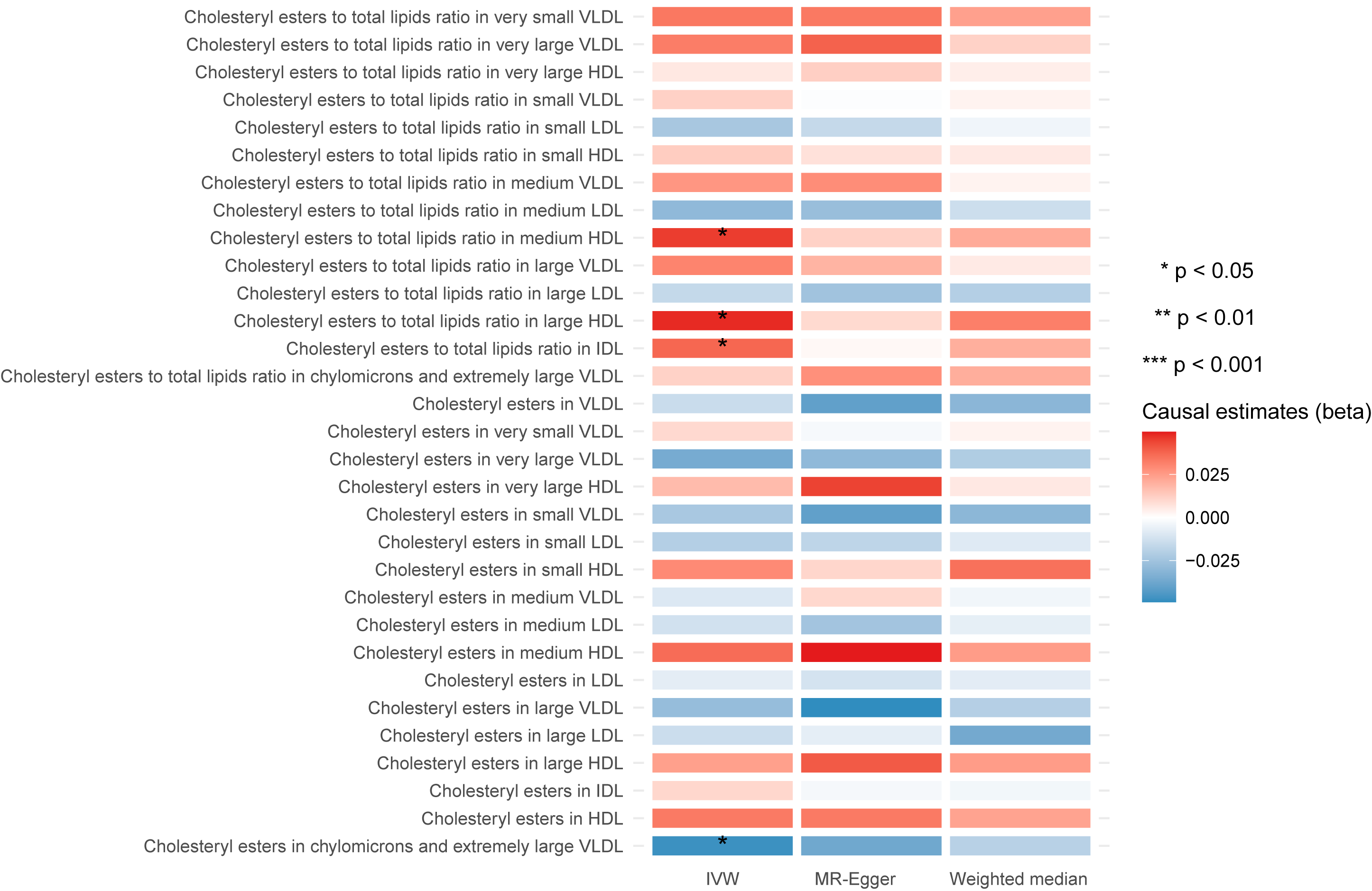

Supplement: Supplementary file 1 — Additional file1: Figure S1. Heatmap showing the causal estimates of triglycerides related traits on facial skin aging in the primary analyses with IVW, MR-Egger, and weighted median methods. Figure S2. Heatmap showing the causal estimates of amino acids on facial skin aging in the primary analyses with IVW, MR-Egger, and weighted median methods. Figure S3. Heatmap showing the causal estimates of cholesterol ester on facial skin aging in the primary analyses with IVW, MR-Egger, and weighted median methods. Figure S4. Heatmap showing the causal estimates of free cholesterol on facial skin aging in the primary analyses with IVW, MR-Egger, and weighted median methods. Figure S5. Heatmap showing the causal estimates of lipoprotein cholesterol on facial skin aging in the primary analyses with IVW, MR-Egger, and weighted median methods. Figure S6: Heatmap showing the causal estimates of small metabolites on facial skin aging in the primary analyses with IVW, MR-Egger, and weighted median methods. Figure S7. Heatmap showing the causal estimates of phospholipids on facial skin aging in the primary analyses with IVW, MR-Egger, and weighted median methods. Figure S8. Heatmap showing the causal estimates of total lipids on facial skin aging in the primary analyses with IVW, MR-Egger, and weighted median methods. Figure S9. Heatmap showing the causal estimates of 123 metabolic traits on facial skin aging in the secondary analyses with IVW, MR-Egger, and weighted median methods. Figure S10. Dot plot of Cook’s distance for the causal effects of degree of unsaturation on facial skin aging with MR-BMA method. Figure S11. Dot plot of Cook’s distance for the causal effects of MUFA on facial skin aging with MR-BMA method. Figure S12. Dot plot of Cook’s distance for the causal effects of PUFA to MUFA ratio on facial skin aging with MR-BMA method. Figure S13. Dot plot of Q-statistics for the causal effects of degree of unsaturation on facial skin aging with MR-BMA method. [file 40246_2023_470_MOESM1_ESM.zip › Supplementary/Supplementary Figure 3. cholesterol Ester.tif]

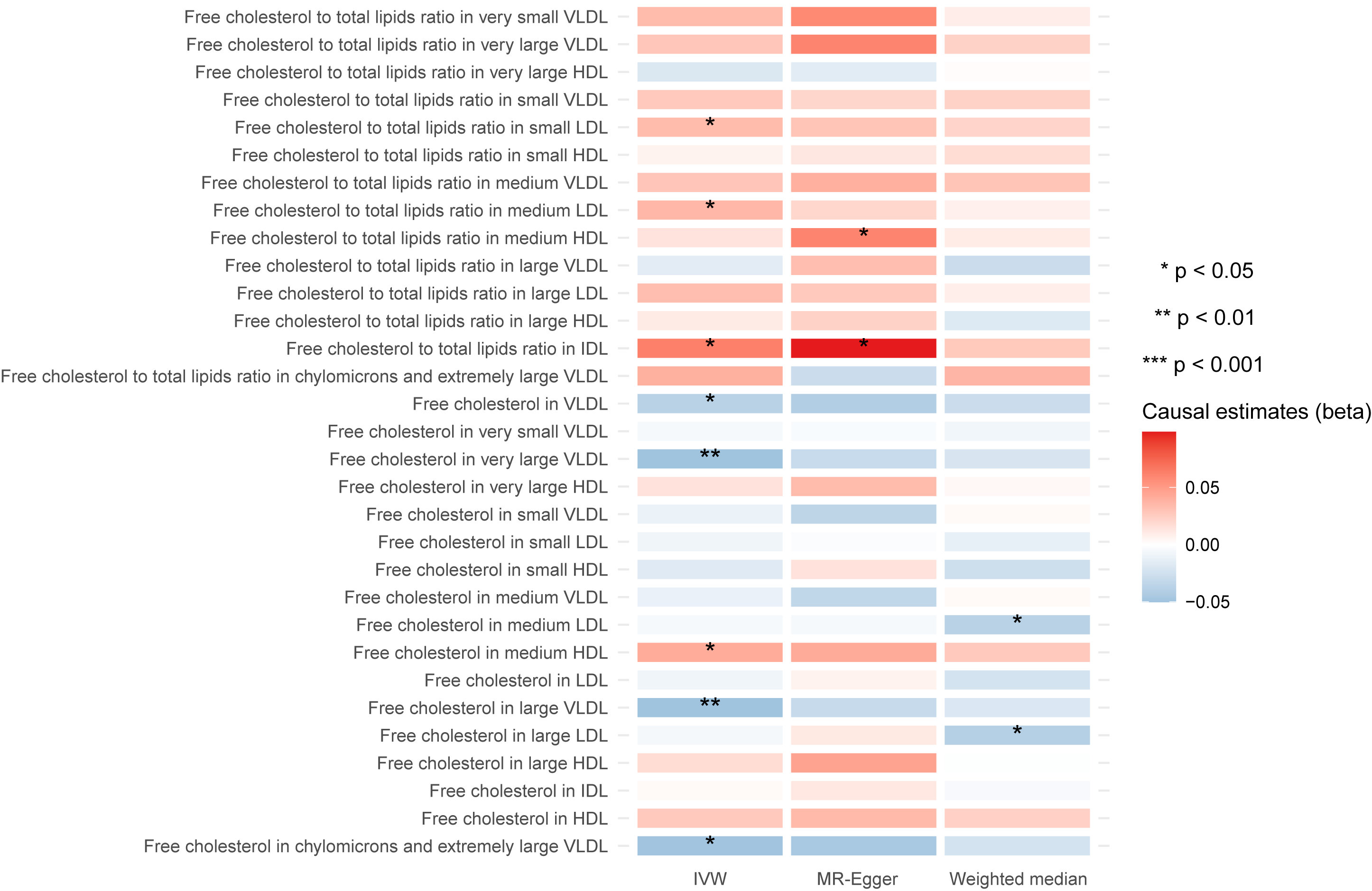

Supplement: Supplementary file 1 — Additional file1: Figure S1. Heatmap showing the causal estimates of triglycerides related traits on facial skin aging in the primary analyses with IVW, MR-Egger, and weighted median methods. Figure S2. Heatmap showing the causal estimates of amino acids on facial skin aging in the primary analyses with IVW, MR-Egger, and weighted median methods. Figure S3. Heatmap showing the causal estimates of cholesterol ester on facial skin aging in the primary analyses with IVW, MR-Egger, and weighted median methods. Figure S4. Heatmap showing the causal estimates of free cholesterol on facial skin aging in the primary analyses with IVW, MR-Egger, and weighted median methods. Figure S5. Heatmap showing the causal estimates of lipoprotein cholesterol on facial skin aging in the primary analyses with IVW, MR-Egger, and weighted median methods. Figure S6: Heatmap showing the causal estimates of small metabolites on facial skin aging in the primary analyses with IVW, MR-Egger, and weighted median methods. Figure S7. Heatmap showing the causal estimates of phospholipids on facial skin aging in the primary analyses with IVW, MR-Egger, and weighted median methods. Figure S8. Heatmap showing the causal estimates of total lipids on facial skin aging in the primary analyses with IVW, MR-Egger, and weighted median methods. Figure S9. Heatmap showing the causal estimates of 123 metabolic traits on facial skin aging in the secondary analyses with IVW, MR-Egger, and weighted median methods. Figure S10. Dot plot of Cook’s distance for the causal effects of degree of unsaturation on facial skin aging with MR-BMA method. Figure S11. Dot plot of Cook’s distance for the causal effects of MUFA on facial skin aging with MR-BMA method. Figure S12. Dot plot of Cook’s distance for the causal effects of PUFA to MUFA ratio on facial skin aging with MR-BMA method. Figure S13. Dot plot of Q-statistics for the causal effects of degree of unsaturation on facial skin aging with MR-BMA method. [file 40246_2023_470_MOESM1_ESM.zip › Supplementary/Supplementary Figure 4. Free cholesterol.tif]

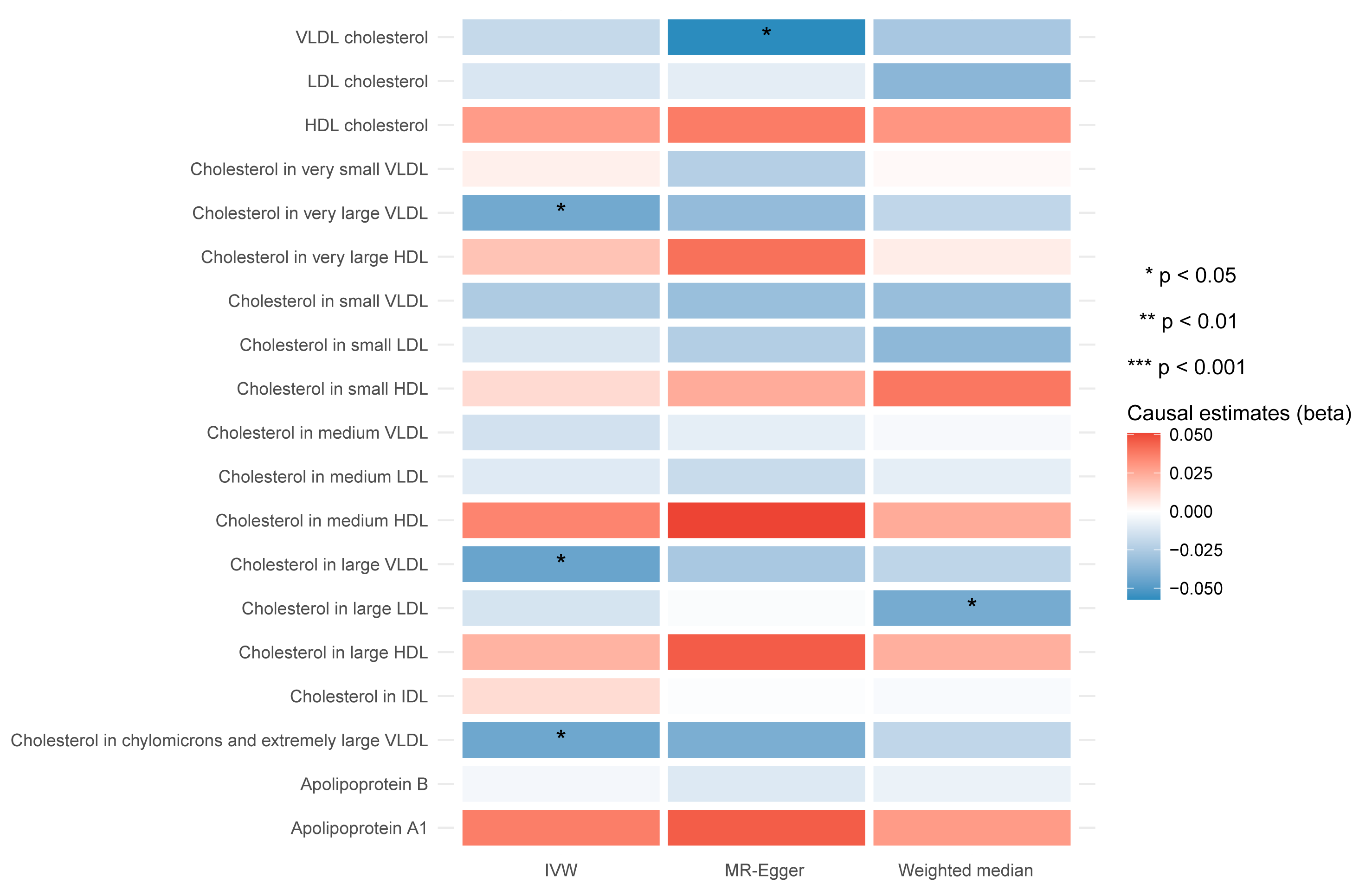

Supplement: Supplementary file 1 — Additional file1: Figure S1. Heatmap showing the causal estimates of triglycerides related traits on facial skin aging in the primary analyses with IVW, MR-Egger, and weighted median methods. Figure S2. Heatmap showing the causal estimates of amino acids on facial skin aging in the primary analyses with IVW, MR-Egger, and weighted median methods. Figure S3. Heatmap showing the causal estimates of cholesterol ester on facial skin aging in the primary analyses with IVW, MR-Egger, and weighted median methods. Figure S4. Heatmap showing the causal estimates of free cholesterol on facial skin aging in the primary analyses with IVW, MR-Egger, and weighted median methods. Figure S5. Heatmap showing the causal estimates of lipoprotein cholesterol on facial skin aging in the primary analyses with IVW, MR-Egger, and weighted median methods. Figure S6: Heatmap showing the causal estimates of small metabolites on facial skin aging in the primary analyses with IVW, MR-Egger, and weighted median methods. Figure S7. Heatmap showing the causal estimates of phospholipids on facial skin aging in the primary analyses with IVW, MR-Egger, and weighted median methods. Figure S8. Heatmap showing the causal estimates of total lipids on facial skin aging in the primary analyses with IVW, MR-Egger, and weighted median methods. Figure S9. Heatmap showing the causal estimates of 123 metabolic traits on facial skin aging in the secondary analyses with IVW, MR-Egger, and weighted median methods. Figure S10. Dot plot of Cook’s distance for the causal effects of degree of unsaturation on facial skin aging with MR-BMA method. Figure S11. Dot plot of Cook’s distance for the causal effects of MUFA on facial skin aging with MR-BMA method. Figure S12. Dot plot of Cook’s distance for the causal effects of PUFA to MUFA ratio on facial skin aging with MR-BMA method. Figure S13. Dot plot of Q-statistics for the causal effects of degree of unsaturation on facial skin aging with MR-BMA method. [file 40246_2023_470_MOESM1_ESM.zip › Supplementary/Supplementary Figure 5. lipoprotein_cholesterol.tif]

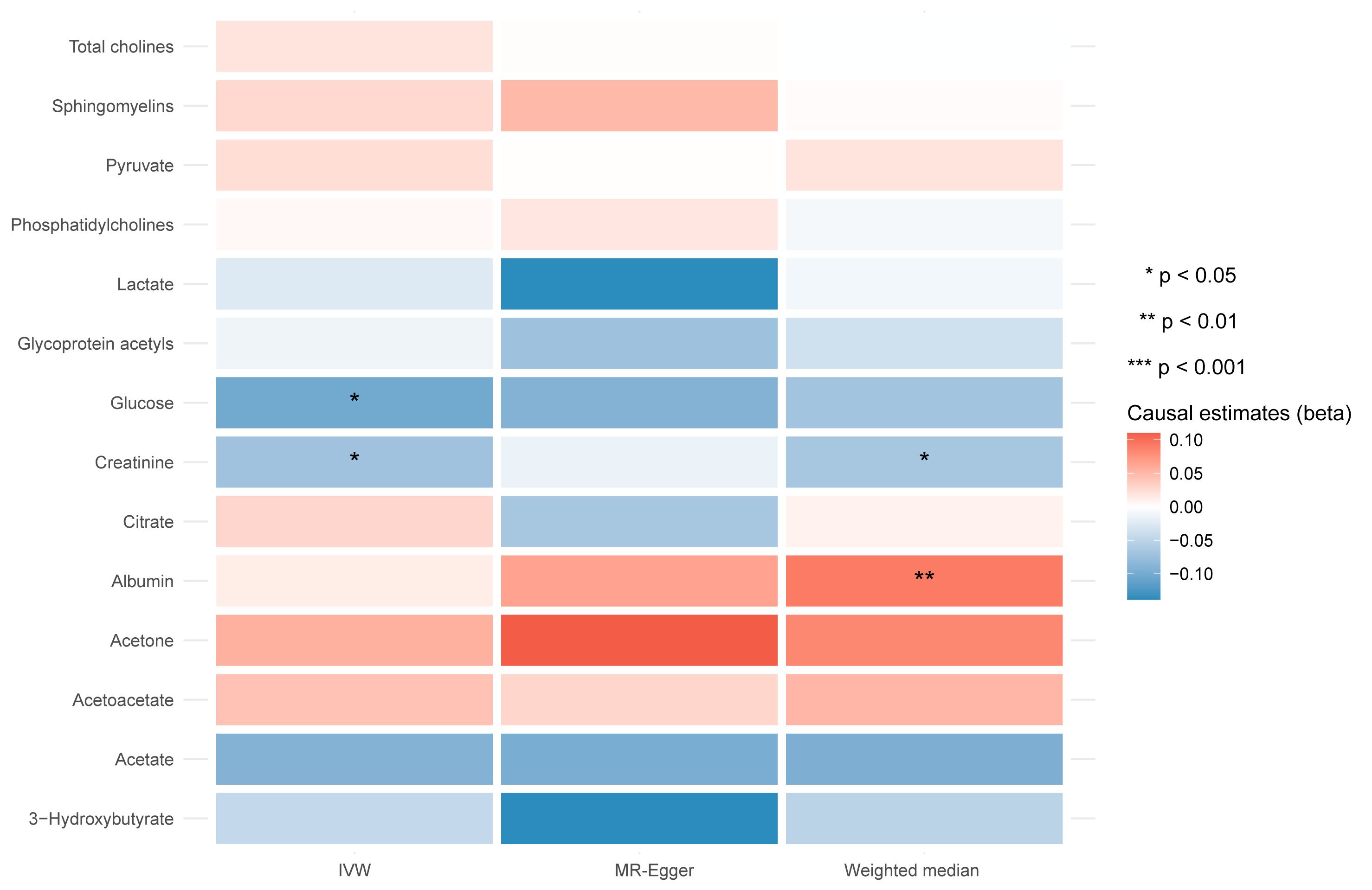

Supplement: Supplementary file 1 — Additional file1: Figure S1. Heatmap showing the causal estimates of triglycerides related traits on facial skin aging in the primary analyses with IVW, MR-Egger, and weighted median methods. Figure S2. Heatmap showing the causal estimates of amino acids on facial skin aging in the primary analyses with IVW, MR-Egger, and weighted median methods. Figure S3. Heatmap showing the causal estimates of cholesterol ester on facial skin aging in the primary analyses with IVW, MR-Egger, and weighted median methods. Figure S4. Heatmap showing the causal estimates of free cholesterol on facial skin aging in the primary analyses with IVW, MR-Egger, and weighted median methods. Figure S5. Heatmap showing the causal estimates of lipoprotein cholesterol on facial skin aging in the primary analyses with IVW, MR-Egger, and weighted median methods. Figure S6: Heatmap showing the causal estimates of small metabolites on facial skin aging in the primary analyses with IVW, MR-Egger, and weighted median methods. Figure S7. Heatmap showing the causal estimates of phospholipids on facial skin aging in the primary analyses with IVW, MR-Egger, and weighted median methods. Figure S8. Heatmap showing the causal estimates of total lipids on facial skin aging in the primary analyses with IVW, MR-Egger, and weighted median methods. Figure S9. Heatmap showing the causal estimates of 123 metabolic traits on facial skin aging in the secondary analyses with IVW, MR-Egger, and weighted median methods. Figure S10. Dot plot of Cook’s distance for the causal effects of degree of unsaturation on facial skin aging with MR-BMA method. Figure S11. Dot plot of Cook’s distance for the causal effects of MUFA on facial skin aging with MR-BMA method. Figure S12. Dot plot of Cook’s distance for the causal effects of PUFA to MUFA ratio on facial skin aging with MR-BMA method. Figure S13. Dot plot of Q-statistics for the causal effects of degree of unsaturation on facial skin aging with MR-BMA method. [file 40246_2023_470_MOESM1_ESM.zip › Supplementary/Supplementary Figure 6. metabolite.tif]

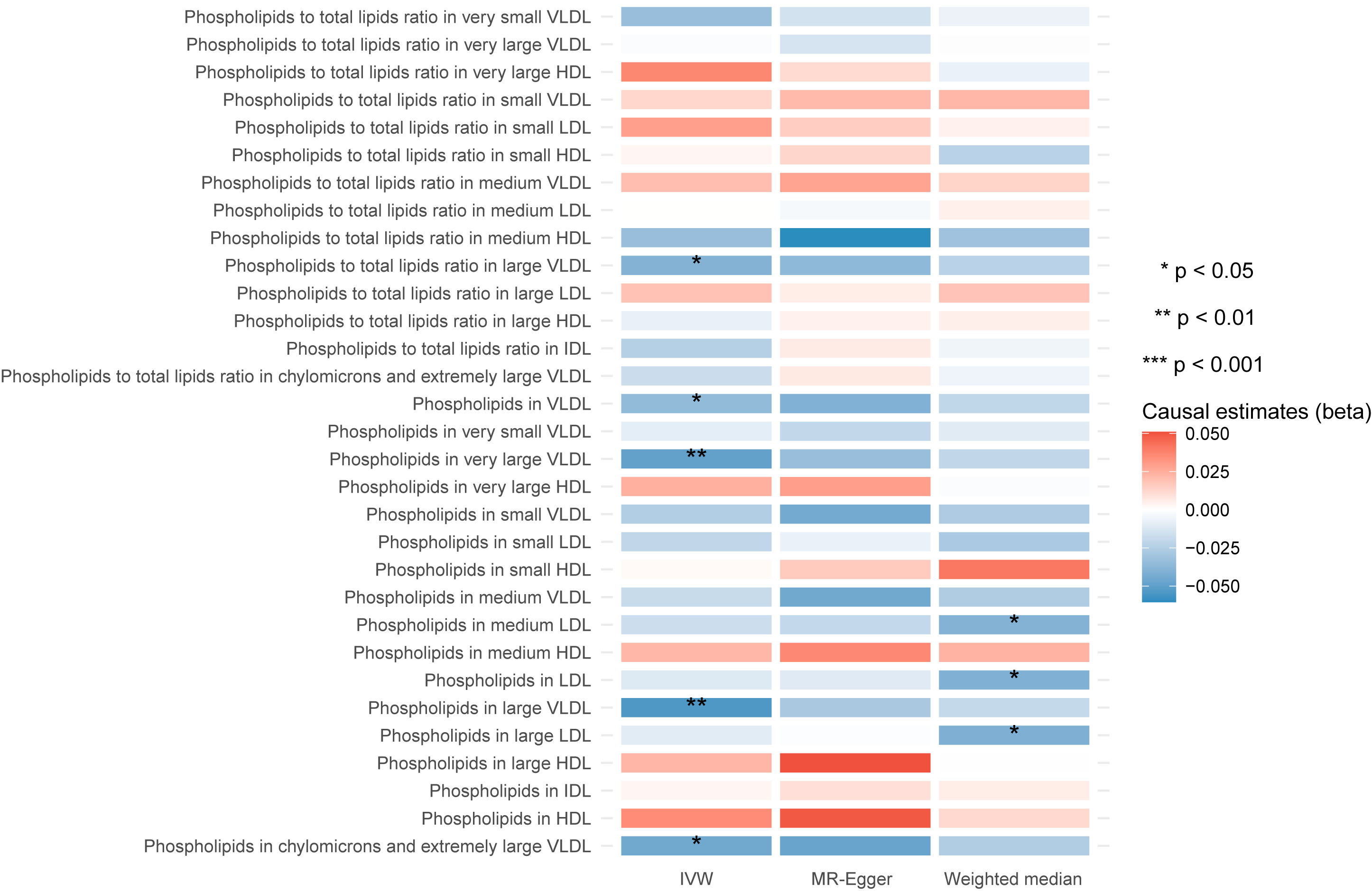

Supplement: Supplementary file 1 — Additional file1: Figure S1. Heatmap showing the causal estimates of triglycerides related traits on facial skin aging in the primary analyses with IVW, MR-Egger, and weighted median methods. Figure S2. Heatmap showing the causal estimates of amino acids on facial skin aging in the primary analyses with IVW, MR-Egger, and weighted median methods. Figure S3. Heatmap showing the causal estimates of cholesterol ester on facial skin aging in the primary analyses with IVW, MR-Egger, and weighted median methods. Figure S4. Heatmap showing the causal estimates of free cholesterol on facial skin aging in the primary analyses with IVW, MR-Egger, and weighted median methods. Figure S5. Heatmap showing the causal estimates of lipoprotein cholesterol on facial skin aging in the primary analyses with IVW, MR-Egger, and weighted median methods. Figure S6: Heatmap showing the causal estimates of small metabolites on facial skin aging in the primary analyses with IVW, MR-Egger, and weighted median methods. Figure S7. Heatmap showing the causal estimates of phospholipids on facial skin aging in the primary analyses with IVW, MR-Egger, and weighted median methods. Figure S8. Heatmap showing the causal estimates of total lipids on facial skin aging in the primary analyses with IVW, MR-Egger, and weighted median methods. Figure S9. Heatmap showing the causal estimates of 123 metabolic traits on facial skin aging in the secondary analyses with IVW, MR-Egger, and weighted median methods. Figure S10. Dot plot of Cook’s distance for the causal effects of degree of unsaturation on facial skin aging with MR-BMA method. Figure S11. Dot plot of Cook’s distance for the causal effects of MUFA on facial skin aging with MR-BMA method. Figure S12. Dot plot of Cook’s distance for the causal effects of PUFA to MUFA ratio on facial skin aging with MR-BMA method. Figure S13. Dot plot of Q-statistics for the causal effects of degree of unsaturation on facial skin aging with MR-BMA method. [file 40246_2023_470_MOESM1_ESM.zip › Supplementary/Supplementary Figure 7. Phospholipids.tif]

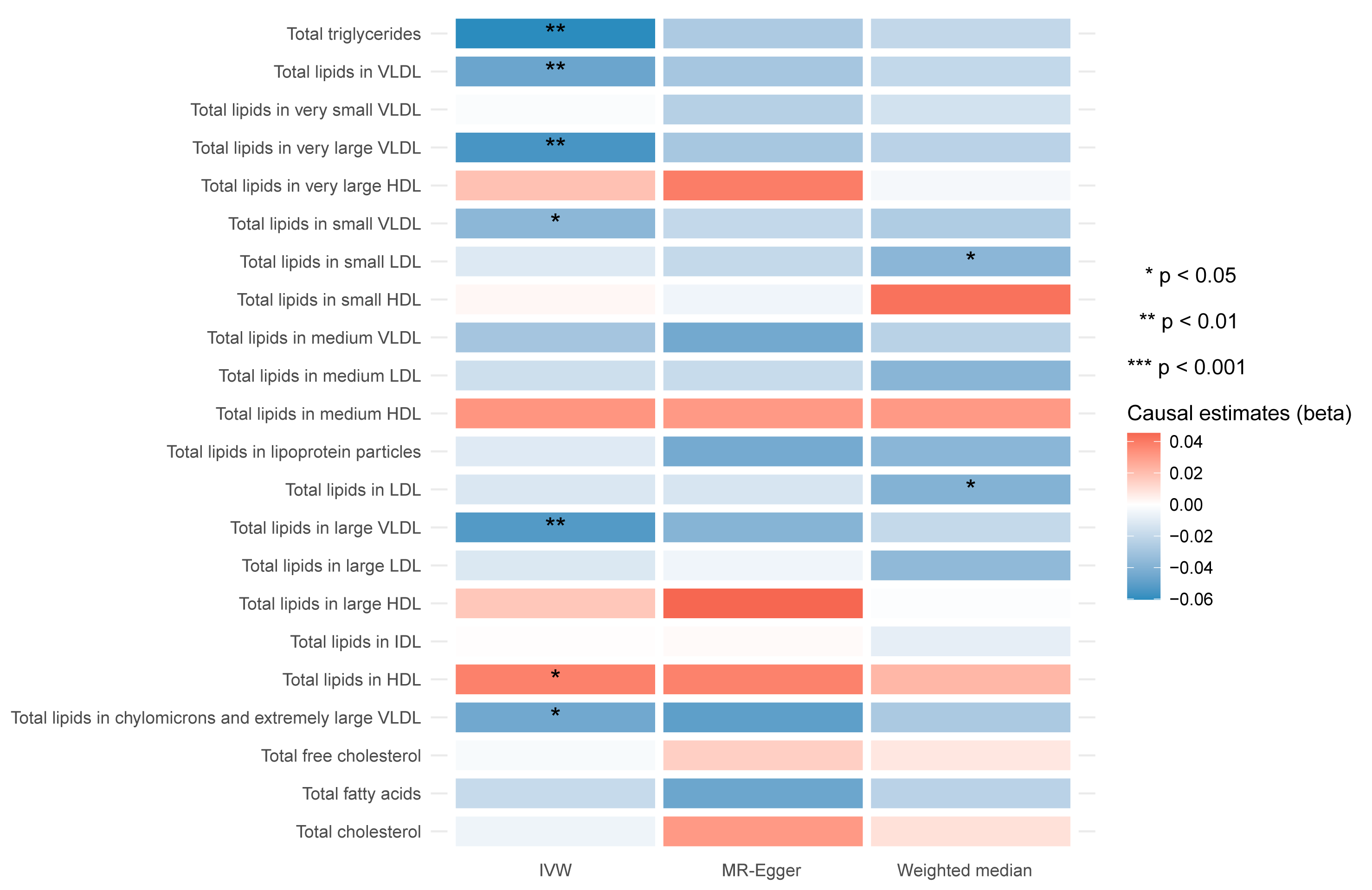

Supplement: Supplementary file 1 — Additional file1: Figure S1. Heatmap showing the causal estimates of triglycerides related traits on facial skin aging in the primary analyses with IVW, MR-Egger, and weighted median methods. Figure S2. Heatmap showing the causal estimates of amino acids on facial skin aging in the primary analyses with IVW, MR-Egger, and weighted median methods. Figure S3. Heatmap showing the causal estimates of cholesterol ester on facial skin aging in the primary analyses with IVW, MR-Egger, and weighted median methods. Figure S4. Heatmap showing the causal estimates of free cholesterol on facial skin aging in the primary analyses with IVW, MR-Egger, and weighted median methods. Figure S5. Heatmap showing the causal estimates of lipoprotein cholesterol on facial skin aging in the primary analyses with IVW, MR-Egger, and weighted median methods. Figure S6: Heatmap showing the causal estimates of small metabolites on facial skin aging in the primary analyses with IVW, MR-Egger, and weighted median methods. Figure S7. Heatmap showing the causal estimates of phospholipids on facial skin aging in the primary analyses with IVW, MR-Egger, and weighted median methods. Figure S8. Heatmap showing the causal estimates of total lipids on facial skin aging in the primary analyses with IVW, MR-Egger, and weighted median methods. Figure S9. Heatmap showing the causal estimates of 123 metabolic traits on facial skin aging in the secondary analyses with IVW, MR-Egger, and weighted median methods. Figure S10. Dot plot of Cook’s distance for the causal effects of degree of unsaturation on facial skin aging with MR-BMA method. Figure S11. Dot plot of Cook’s distance for the causal effects of MUFA on facial skin aging with MR-BMA method. Figure S12. Dot plot of Cook’s distance for the causal effects of PUFA to MUFA ratio on facial skin aging with MR-BMA method. Figure S13. Dot plot of Q-statistics for the causal effects of degree of unsaturation on facial skin aging with MR-BMA method. [file 40246_2023_470_MOESM1_ESM.zip › Supplementary/Supplementary Figure 8. total lipid.tif]

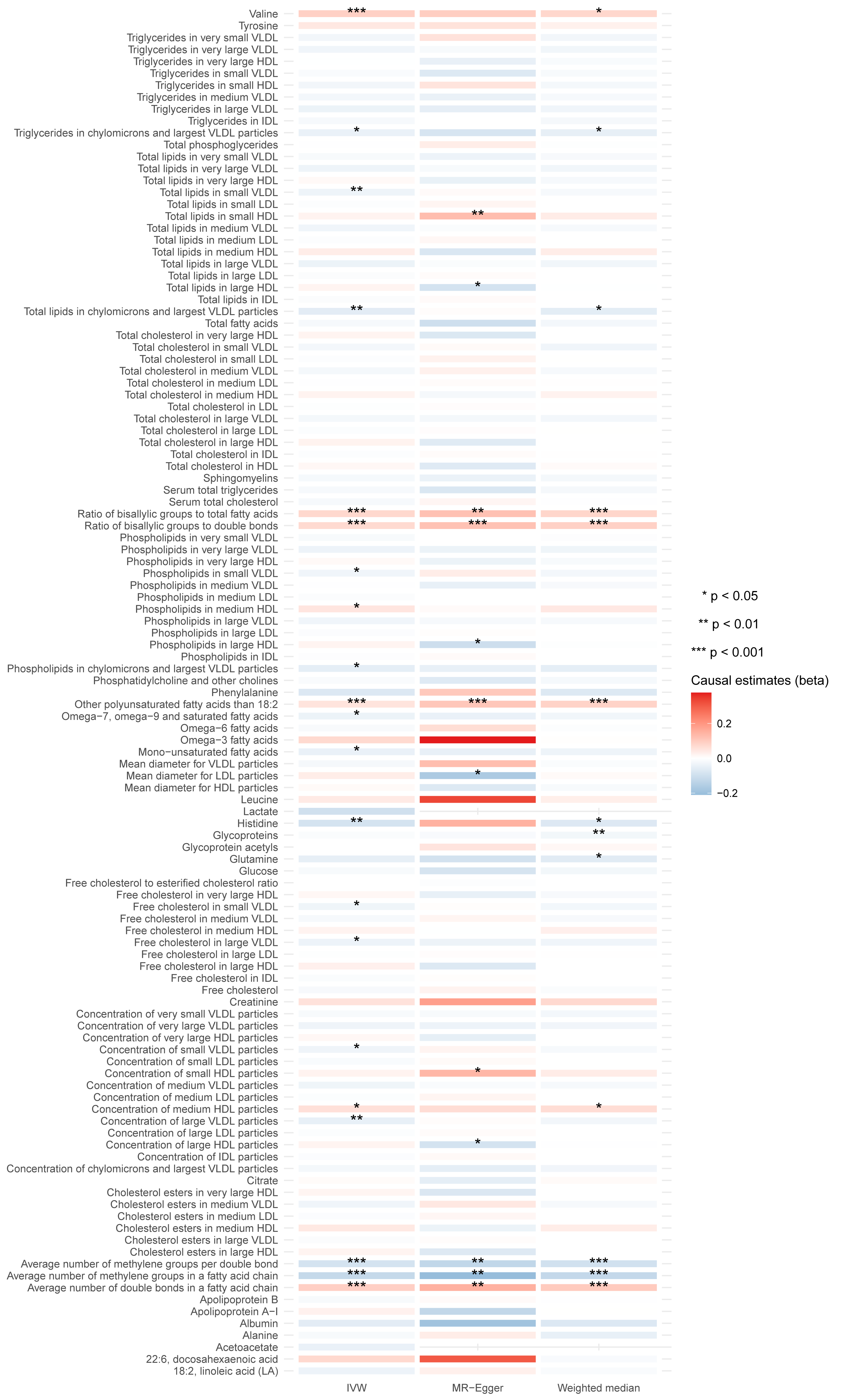

Supplement: Supplementary file 1 — Additional file1: Figure S1. Heatmap showing the causal estimates of triglycerides related traits on facial skin aging in the primary analyses with IVW, MR-Egger, and weighted median methods. Figure S2. Heatmap showing the causal estimates of amino acids on facial skin aging in the primary analyses with IVW, MR-Egger, and weighted median methods. Figure S3. Heatmap showing the causal estimates of cholesterol ester on facial skin aging in the primary analyses with IVW, MR-Egger, and weighted median methods. Figure S4. Heatmap showing the causal estimates of free cholesterol on facial skin aging in the primary analyses with IVW, MR-Egger, and weighted median methods. Figure S5. Heatmap showing the causal estimates of lipoprotein cholesterol on facial skin aging in the primary analyses with IVW, MR-Egger, and weighted median methods. Figure S6: Heatmap showing the causal estimates of small metabolites on facial skin aging in the primary analyses with IVW, MR-Egger, and weighted median methods. Figure S7. Heatmap showing the causal estimates of phospholipids on facial skin aging in the primary analyses with IVW, MR-Egger, and weighted median methods. Figure S8. Heatmap showing the causal estimates of total lipids on facial skin aging in the primary analyses with IVW, MR-Egger, and weighted median methods. Figure S9. Heatmap showing the causal estimates of 123 metabolic traits on facial skin aging in the secondary analyses with IVW, MR-Egger, and weighted median methods. Figure S10. Dot plot of Cook’s distance for the causal effects of degree of unsaturation on facial skin aging with MR-BMA method. Figure S11. Dot plot of Cook’s distance for the causal effects of MUFA on facial skin aging with MR-BMA method. Figure S12. Dot plot of Cook’s distance for the causal effects of PUFA to MUFA ratio on facial skin aging with MR-BMA method. Figure S13. Dot plot of Q-statistics for the causal effects of degree of unsaturation on facial skin aging with MR-BMA method. [file 40246_2023_470_MOESM1_ESM.zip › Supplementary/Supplementary Figure 9.tif]
